# Linking musical metaphors and emotions evoked by the sound of classical music

**DOI:** 10.1177/0305735621991235

**Published:** 2021-03-24

**Authors:** Simon Schaerlaeken, Donald Glowinski, Didier Grandjean

**Affiliations:** 1 Neuroscience of Emotion and Affective Dynamics Lab, Department of Psychology and Educational Sciences, University of Geneva, Geneva, Switzerland; 2 Swiss Center for Affective Sciences, University of Geneva, Geneva, Switzerland

**Keywords:** metaphors, emotions, music, meaning, GEMS, GEMMES

## Abstract

Musical meaning is often described in terms of emotions and metaphors. While many theories encapsulate one or the other, very little empirical data is available to test a possible link between the two. In this article, we examined the metaphorical and emotional contents of Western classical music using the answers of 162 participants. We calculated generalized linear mixed-effects models, correlations, and multidimensional scaling to connect emotions and metaphors. It resulted in each metaphor being associated with different specific emotions, subjective levels of entrainment, and acoustic and perceptual characteristics. How these constructs relate to one another could be based on the embodied knowledge and the perception of movement in space. For instance, metaphors that rely on movement are related to emotions associated with movement. In addition, measures in this study could also be represented by underlying dimensions such as valence and arousal. Musical writing and music education could benefit greatly from these results. Finally, we suggest that music researchers consider musical metaphors in their work as we provide an empirical method for it.

How do we understand music? It has been argued that people have a “knowledge instinct,” an innate need to understand the world by building up and representing complex structures and summarizing the pieces of knowledge acquired along the way (Perlovsky, 2007). By putting parts together, the human brain creates meaning as it experiences the world. Like language, music has a hierarchically connected structure of smaller components that can be linked to one another in order to extract a meaning that unfolds over time (Cooper & Meyer, 1960; Krumhansl, 1990; Levitin & Menon, 2003, 2005; Patel, 2003). Such a meaning can result from conceptual assignments that can be cross-domain if we “hear music as . . .” (Larson, 2012). In this process, metaphors are an important cross-domain mapping that helps us understand the musical experience (Scruton, 1999). Metaphors, as described by Lakoff and Johnson’s theory, are viewed as a conceptual process in which we understand one concept in terms of another (M. Johnson, 1987; Lakoff & Johnson, 1980). They often rely on image-schematic structures grounded in embodied experiences to create the necessary mappings (Bonde, 2007; Zbikowski, 2008). Similarly grounded in embodied experience are the creation of meaning and the use of conceptual knowledge (Aksnes, 2000; Borgo, 2004; Chuck, 2004; Cox, 2001; M. L. Johnson, 1997; Walker, 2000). They seem to result from the reactivation of modality-specific areas such as motor and sensory areas (Barsalou, 2005). These areas, interconnected when performing an action or sensory perception, can be reactivated in the context of conceptual tasks, thereby providing an embodied representation of conceptual knowledge. As children grow up, physical experiences are transformed and stored in the human mind to be at the center of conceptual knowledge. Piaget proposed a theory for cognitive development explaining that children construct knowledge and understanding of the world by coordinating experiences from physical interaction with objects (e.g., stepping, grasping, and sucking; Piaget & Inhelder, 1969). Several central concepts such as height, path, containment focus on this implicitly embodied learning. In this context, metaphors are seen as the basic structure of understanding in that such concepts are used to help the individual understand his environment (Lakoff, 1993).

Many metaphors have been suggested for music. Concepts like time (Epstein, 1995), space (Bonde, 2007), movement (M. L. Johnson & Larson, 2003; Rothfarb, 2002; Scruton, 1999), and force (Larson, 2012) are consistently applied to the musical field. For example, pitch is described in terms of height (high or low) in Western culture. But different cultures use different combinations of terms to represent pitch, such as light/heavy for the Kpelle people in Liberia (Stone, 1981) and young/old for Suya people in the Amazon Basin (Zbikowski, 1998). It has been shown that language plays a causal role in the design of nonlinguistic representations of pitch, for example, when two populations with two different representations of pitch are asked to exchange them (Dolscheid, Shayan, Majid, & Casasanto, 2013). Clifton (1983) even wrote in his book: “The most central and universal characteristics of music (patterns of tension and release, the gestural, the sensuous) are meaningful only because they are known by the body. Music does not arise from an objective examination of syntactical or formal functions, but from bodily complicity with sounds” (Clifton, 1983; p. 279). Musicological writings today are dominated by the idea of music as a continuous, unidirectional forward movement through space (Cumming, 2000). Recently, a series of three studies attempted to capture the most widespread categories of metaphors associated with Western classical music (Schaerlaeken, Glowinski, Rappaz, & Grandjean, 2019). This led to the creation of the Geneva Musical Metaphors Scale (GEMMES), which consists of five subscales that can be linked to the most commonly used or relevant families of musical metaphors: “Flow,” “Force,” “Interior,” “Movement,” and “Wandering.”

Another type of meaning extracted from music is centered around emotions and affective processes. It has been argued that the ability of music to evoke emotions (Dowling & Harwood, 1986) is one of the main reasons why people engage with it (Juslin & Laukka, 2004) and its primary purpose (Cooke, 1959). It is known that music artists are able to convey certain emotions (e.g., sadness, anger, happiness, and fear) to the audience (Behrens & Green, 1993; Gabrielsson, 1995; Gabrielsson & Juslin, 1996; Juslin & Madison, 1999). Traditional models of emotions like basic emotions (Ekman, 1992) and dimensional models like valence and arousal (Russell, 1980) have also been used to describe the perception of emotions in pieces of music. In a review, Eerola and colleagues highlighted that 70% of 251 studies of music used variants of the discrete or dimensional emotion model (Eerola & Vuoskoski, 2013). When compared, the main difference between the two models was the poorer resolution of the discrete model when characterizing emotionally ambiguous pieces (Eerola & Vuoskoski, 2011). In fact, music can evoke a variety of different experiences in the listener, from sheer excitement, chills, and some “basic emotions” to “more complex” and “mixed” emotions (e.g., nostalgia and pride; Juslin, 2011; Juslin, Liljestrom, Laukka, Vastfjall, & Lundqvist, 2011). In order to characterize musical emotions in a more differentiated way, the Geneva Emotion Musical Scale (GEMS; Zentner, Grandjean, & Scherer, 2008) tried to report all relevant musical emotions by examining the relevance of emotional terms for different musical genres. However, by pointing out some of the weaknesses of the GEMS, such as the difficulty in interpreting or differentiating some factors (e.g., “wonder” and “transcendence”), researchers have found that using a dimensional model could most often be the most reliable or preferred method of collecting and presenting musical emotion data (Vuoskoski & Eerola, 2011), even if such an approach dramatically reduces the wealth of the listeners’ emotional experiences.

In addition, it has been suggested that the experience of musical emotions is determined by emotion-specific complex acoustic patterns that can be also found in vocal expressions (Juslin & Laukka, 2003). The relationships between emotion and acoustic features are only probabilistic and can best be understood as correlative (Juslin, 2000). Several associations have already been highlighted, such as sadness with slow tempo and legato articulation, while happiness shows a faster tempo and staccato articulation (Gabrielsson and Juslin (1996); see meta-analysis (Juslin & Laukka, 2003). Emotions experienced by the listeners are supported not only on the acoustic and musical structural features, but are also influenced by a variety of parameters that relate to listener characteristics and states, cultural contexts, and the performance of the musicians (e.g., Gabrielsson, 2001; Scherer & Zentner, 2001; Scherer, Zentner, & Schacht, 2001). Entrainment, the tendency to synchronize with the beat, also seems to play an important role in the development of emotions when listening to music (Juslin & Västfjäll, 2008; Labbé & Grandjean, 2014). A powerful rhythm in the music could affect the listener’s internal body rhythm (e.g., heart rate). The adjusted heart rate can then spread to other components of emotions, such as feelings, through proprioceptive feedback (Juslin, 2013). All in all, musical emotions can be evoked from the sounds of a piece of music as a kind of basic building block, but they can also be conveyed through schematic expectations and knowledge of musical forms (Davies, 1994; Levinson, 1980).

In order to capture the musical meaning, we believe that exploring together the emotional and metaphorical content of a piece of music can help create a more complete picture. Metaphors and emotions have always been closely linked in this context. Emotional language is dominated by metaphorical expressions (e.g., “I’m feeling down”; Kövecses, 2008; Lakoff & Johnson, 1980). Similarly, of course, metaphors are used to describe music, even at a young age (Flowers & Wang, 2002), because describing subjective experiences (e.g., emotions or listening to music) with non-metaphorical or analogical reasoning is often impossible (Lakoff & Johnson, 1999). It was shown that metaphors in turn influence cognition and sensory experiences (see for a review Landau, Meier, & Keefer, 2010). In the context of music, they represent a platform for the common understanding of the affective quality of music and can enable the transition from emotion perception to emotion induction (Pannese, Rappaz, & Grandjean, 2016). In a study, when listening to music with different emotional characteristics (e.g., sad and happy), the participants showed distortions in the assessment of the brightness of gray squares according to the metaphors “positive is light” and “negative is dark” (Bhattacharya & Lindsen, 2016). In another study, in collecting narratives describing the experience of sad music, it turned out that the participants had a rich vocabulary of metaphors focused on movement and space to describe their affective experiences (Peltola & Saresma, 2014). Movement-based metaphors (e.g., bouncing and flowing) are also used to shape the musical performance, as they are often used in music lessons to provide a critical link between the music being presented and the emotion felt (Woody, 2002). Outside of the music world, positive and negative life experiences were also implicitly associated with schematic representations of the upward and downward movements (Casasanto & Dijkstra, 2010). Similarly, the memory of the location of an emotional image can be influenced by the connection between spatial metaphors and emotions, showing that positive emotions are stored as relatively higher than negative ones, which supports the common understanding of the metaphors “good = high” and “bad = down” (Elizabeth Crawford, Margolies, Drake, & Murphy, 2006). Such metaphors, highlighted in Lakoff’s and Johnson’s (1980) theory of conceptual metaphors, are striking examples of the connection between emotions and embodied experiences such as spatial orientation. It can also be found in metaphors like “I am on top of the situation.” In the music world, however, this precise metaphor is not always perfectly represented. While pitch is associated with height in Western music, “up” is not necessarily “more” or “good” when it comes to music (Eitan & Timmers, 2010).

Several theoretical works support links between the acoustical and musical structures, emotions, and metaphors: the BRECVEMA (Juslin, 2013), the extra-musical meaning (Koelsch, 2011), the conceptual blending (Fauconnier & Turner, 2008), and the hierarchical system of six contextual constraints to build meaning (Antović, 2018) (Online Supplemental Material A). However, empirical evidence for these connections is rather sparse. To the best of our knowledge, no comprehensive study has attempted to combine musical emotions, metaphors, and acoustic features. At this point, empirical data is needed to reveal patterns of musical meaning. We believe that both emotions and metaphors are interrelated and could be based on the embodied experiences that relate to specific perceptual features on an acoustic and musical level. To fill the gap in our understanding of musical meaning, we aim to test how metaphors relate to emotions, perceptual features (both musical and more basic perceptual auditory aspects), and acoustic features.

## Method

### Participants

We recruited 162 participants for this study (65 females, M=28.4 years, SD=12.2 ). This number of participants was based on a priori power analysis using data on metaphors from our previous studies to achieve a similar level of significance when comparing the ratings (Schaerlaeken et al., 2019). All participants were French-speaking Europeans with normal, self-reported listening skills. 85 participants were characterized as musicians using a demographic questionnaire asking them if they considered themselves as musicians with more than 5 years of instrument practice and regular practice per week. The study was approved by the Ethics Committee of the Department of Psychology and Education at the University of Geneva.

### Materials

**Figure 1.**
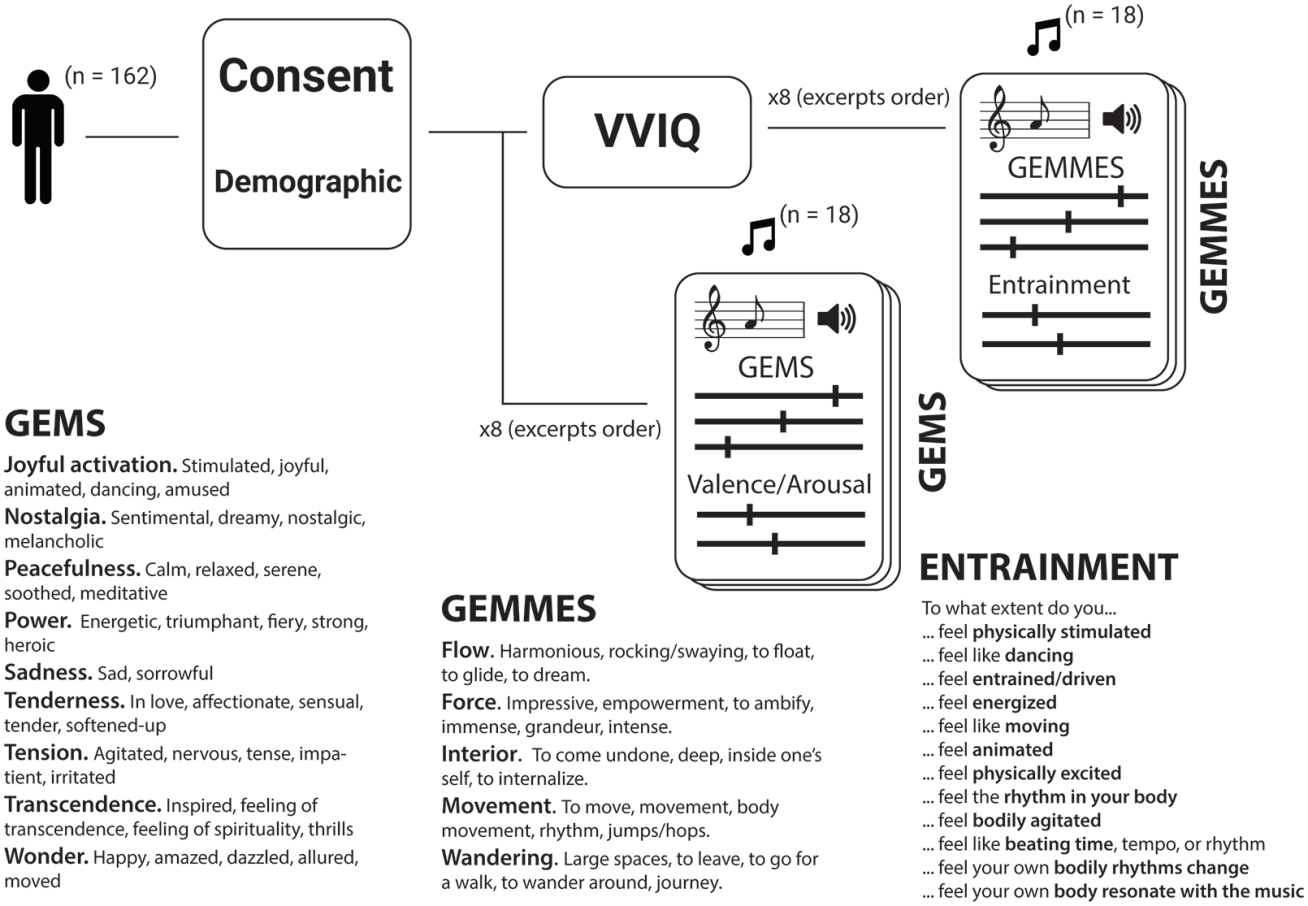
Diagram of the Procedure. All Scales Used in this Study Are Detailed, Introducing the Constituent Terms for Each Item.

Based on a pilot study (Online Supplemental Material B), 18 music excerpts were selected, 2 for each emotion of the 9 GEMS scales. During the study, these excerpts were rated based on how many participants knew them. If the participants did not know them, this would ensure that the metaphors and emotions gathered in these excerpts were not the result of an episodic memory that was irrelevant to the music itself. To explore our main research question, we used different scales to measure metaphors, emotions, multiple perceptual features, and acoustic measures (Figure 1). The musical emotions were rated on two different scales: the GEMS (Zentner et al., 2008) and the dimensional model of valence and arousal (Russell, 1980). The GEMS consists of nine different subscales, representing distinct categories of emotions: “Joyful activation,” “Nostalgia,” “Peacefulness,” “Power,” “Sadness,” “Tenderness,” “Tension,” “Transcendence,” and “Wonder.” The dimensional model consists of two different subscales: “Valence,” in which an emotion is described as positive or negative, and “Arousal,” in which an emotion is described as more or less intense. The metaphors were assessed with the GEMMES (Schaerlaeken et al., 2019) which consists of five subscales: “Flow,” “Force,” “Interior,” “Movement,” and “Wandering.” The entrainment caused by the music was also assessed with the Musical Entrainment Questionnaire (Labbé & Grandjean, 2014). It contained 12 items that evaluate subjective musical entrainment (e.g., “Can you feel the beat?,” “Do you want to dance?”). Finally, the vividness of the imagination of the participants was assessed using the Vivid Visual Imagery Questionnaire (VVIQ), which was translated into French by a group of bilingual speakers (Marks, 1973). This questionnaire asked the participant to imagine different scenes and evaluate how vivid their mental images are. All questionnaires were administered in French.

### Procedure

The study was promoted on social media, on campus through flyers, and through email lists. Participants received an email confirming their registration with a brief description and a link to the entire study, which was managed online through Qualtrics (Qualtrics, Provo, UT). After signing an online consent form and completing a demographic questionnaire, participants were evenly divided between only one of two types of questionnaires: the GEMS and the GEMMES. These two key self-report measures were therefore administered as an independent group measure. Separating participants into two groups allowed us to ensure that the ratings on one scale would not impact the ratings on the other. We believe that such separation benefited our study by avoiding the impact of episodic memory and previous exposition of musical excerpts compared with what a repeated measures design could have achieved. For both types of questionnaires, participants were asked to describe each of the 18 excerpts (Figure 1). Participants had to listen to the entirety of each excerpt of music ( $M_{duration}=99.9$ s, SD=30.8 s) and could fill out the questionnaire at the same time. Wearing headphones was recommended. They were asked to rate how much they experienced each of the nine emotions of the GEMS or each of the five metaphors of the GEMMES on a Likert-type scale (1 to 8, 1 being *not at all* and 8 being *very much*). We had provided further description for each emotion/metaphor based on the information that appears in the respective publications for GEMS/GEMMES (Schaerlaeken et al., 2019; Zentner et al., 2008). For the group of participants who rated emotions, they were also asked to rate the excerpts on both “Valence” and “Arousal” subscales (for “Valence”: −4 to 4, −4 being *negative* and 4 being *positive*; for “Arousal,” 1 to 8, 1 being *not arousing at all* and 8 being *very arousing*). For the other group that rated metaphors, they were also instructed to fill out the Musical Entrainment Questionnaire (Labbé & Grandjean, 2014). The group that evaluated metaphors also had to fill out a VVIQ at the start of the experiment to assess their vivid imagination (Marks, 1973). For both types of questionnaires, the study took approximately 50 min to complete. We offered a financial reward upon completion (either CHF 10 or an Amazon gift card of a similar amount).

### Statistical analyses

We computed a set of acoustical and musical features on all musical excerpts using the MIR toolbox (Lartillot & Toiviainen, 2007). This set of 36 features had been used in previous studies and is adapted for studying musical acoustic features (Eliard, 2017). In addition, we obtained a number of perceptual features for each extract as part of another separate experiment (Aljanaki & Soleymani, 2018). The set contained seven characteristics: articulation, atonality, dissonance, melody, mode, rhythm complexity, and rhythm stability (cf. Aljanaki and Soleymani [2018], for methodology). We performed three principal component analyses (PCA) in order to reduce the dimensionality of our models (Online Supplemental Material C).

After transforming the data into a binary measure (Online Supplemental Material D), we calculated generalized linear mixed models (GLMMs) to estimate the percentage of positive binary ratings of each scale based on a variety of different fixed effects. GLMMs use the modeling of random effects to improve the accuracy of the model and enable the computation of models with abnormal distribution. We calculated our models with a binomial distribution. The random intercept effects in our models encapsulated the variability related to each participant and each musical excerpt. We used a step-up strategy when building the model to compare the different combinations of fixed effects. This comparison was calculated using chi-square difference tests between different models of increasing complexity to examine the contribution of the explained variance for each variable and their interactions. We report on the effect sizes according to the approach of Nakagawa and Schielzeth (2013), which is implemented in the “MuMIn” R package (Nakagawa & Schielzeth, 2013).^1^ Each excerpt was characterized by different labels that were later used as fixed effects. By design, each excerpt represented a single emotion from the GEMS. In addition, each extract could be designated high or low based on its calculated value under any of the following conditions: the components of the acoustic features, the components of the perceptual features, the subjective entrainment component, and the perceived emotions. This distinction was based on the normalized max–min mean score for each condition.

Finally, we calculated the correlations between the various scales and components. Since different participants rated the scales using the two different questionnaires (metaphors vs. emotions), no simple correlations could be calculated. Therefore, we randomly rearranged a thousand times the order of the participants’ ratings for each scale and calculated the Spearman’s correlations between the individual elements (emotions, metaphors, subjective entrainment, acoustic, and perceptual components). Then we extracted the mean correlation for each pair from the normal distribution produced by a thousand random permutations. We used these correlation values as inputs to a multidimensional scaling (MDS) method. The results of the MDS were then clustered using a k-means clustering approach with the city block method to group emotions, metaphors, and features through meaningful associations. Original data can be found at https://github.com/simonschaerlaeken/GEMMES.git

## Results

The aim of this study was to describe the relationships between the musical metaphors reported in the GEMMES, the musical emotions assessed with the GEMS, and a variety of musical descriptors, including acoustic parameters, perceptual features, and subjective entrainment. First, we created profiles for each metaphor that characterize its association with all other descriptors. Second, by calculating the correlation between the various scales, we were able to perform a MDS to examine the global structure of the various measures.

### Comparing groups of participants

Using permutation testing, we found no significant differences between the ratings for both musicians and non-musicians for each GEMS subscale as (Zrange=[–1.00,1.52], $p_{range}=[0.13,0.79]),$ for each GEMMES subscale $(Z_{range}=[-1.19,0.59],p_{range}=[0.23,0.75]),$ and for Valence and Arousal $(Z_{range}=[0.49,1.14],p_{range}=[0.25,0.62]),$ (Online Supplemental Material E). Half of all the participants (N=81) received the GEMMES questionnaire. They had to answer the VVIQ first to assess their ability to imagine mentally vivid images (Marks, 1973). They were divided into two groups: participants with high vivid imagination (upper 25%) and participants with low vivid imagination (lower 25%), based on the number of points obtained in the questionnaire according to the guideline for the VVIQ (Marks, 1973). Some metaphor (“Flow,” “Interior,” and “Wandering”) ratings were significantly affected by how vivid the imagination of our participants was and were rated significantly higher for the group with high vivid imagination as $\begin{array}{c} (Z{\;}_{Flow}\;=-2.21\;,\;\;\;p_{Flow}=0.044\;;\;\;\;Z_{\;Force}=-0.41\;,\;\;p{\;}_{Force}=0.679\;;\;\;\;Z{\;}_{Interior}\;=-3.25\;,\;\;\;p{\;}_{Interior}\;=0.005\;;\\ Z_{Movement}=-1.63,p_{Movement}=0.128;Z_{Wandering}=-2.32,p_{Wandering}=0.044). \end{array}$ Only the metaphor families “Force” and “Movement” were not rated significantly higher for participants with a high imagination than participants with a low imagination. In the raw data, we observed that zeros accounted for around 32% of responses to the GEMS, 16% to the GEMMES, and 6% to Valence and Arousal. Finally, in the 18 musical excerpts, the reliability of the ratings on each item ranged from standardized $\alpha_{Cronbach}=.70$ to .90 (Online Supplemental Material F).

### Relating metaphors to emotions and perceptual features

**Figure 2.**
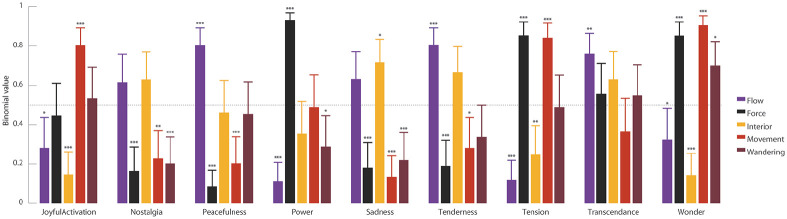
Estimated Binary Ratings GEMMES Based on the Attributed Affective Content of the Musical Excerpts. The Dotted Horizontal Line at 0.5 Symbolized the Chance Level of Drawing From a Binary Set. Values Are Tested to be Significantly Different From This Value. All Contrasts Are FDR-Corrected [* p<.05 , ** p<.01 , *** p<.001 ].*Note.* Please refer to the online version of the article to view the figure in colour.

After we checked that the participants assigned each extract to the correct emotion (Online Supplemental Material G), we assigned each extract to the metaphor scales. We reported that a model that included the interaction between the selected metaphors and the emotion labels and the main effects associated with them outperformed a model with only the main effects ( $\chi^{2}(47,N=81)=1710.3,\;\;\;p<.001,\;\;\;R_{m}^{2}=.27,\;\;\;R_{c}^{2}=.45,\;\;\;AIC_{GEMMES*ExcerptGEMS}=7,559,\;\;\;AIC_{GEMMES+ExcerptGEMS}=9,205,$ $BIC_{GEMMES*ExcerptGEMS}=7,882,$ $BIC_{GEMMES+ExcerptGEMS}=9,308$ ). Participants’ ratings were higher for the metaphor “Movement” in excerpts labeled as “Joyful Activation,” higher for the metaphor “Force” in excerpts labeled as “Power,” and higher for both in excerpts labeled as “Tension” and “Wonder” (Figure 2, Online Supplemental Material I). The ratings for the metaphor “Flow” in excerpts labeled as “Peacefulness,” “Tenderness,” and “Transcendence” were higher. While the ratings for the metaphor “Interior” were only higher for excerpts labeled as “Sadness,” the ratings for the metaphor “Wandering” were only higher for the excerpts labeled as “Wonder.”

**Figure 3.**
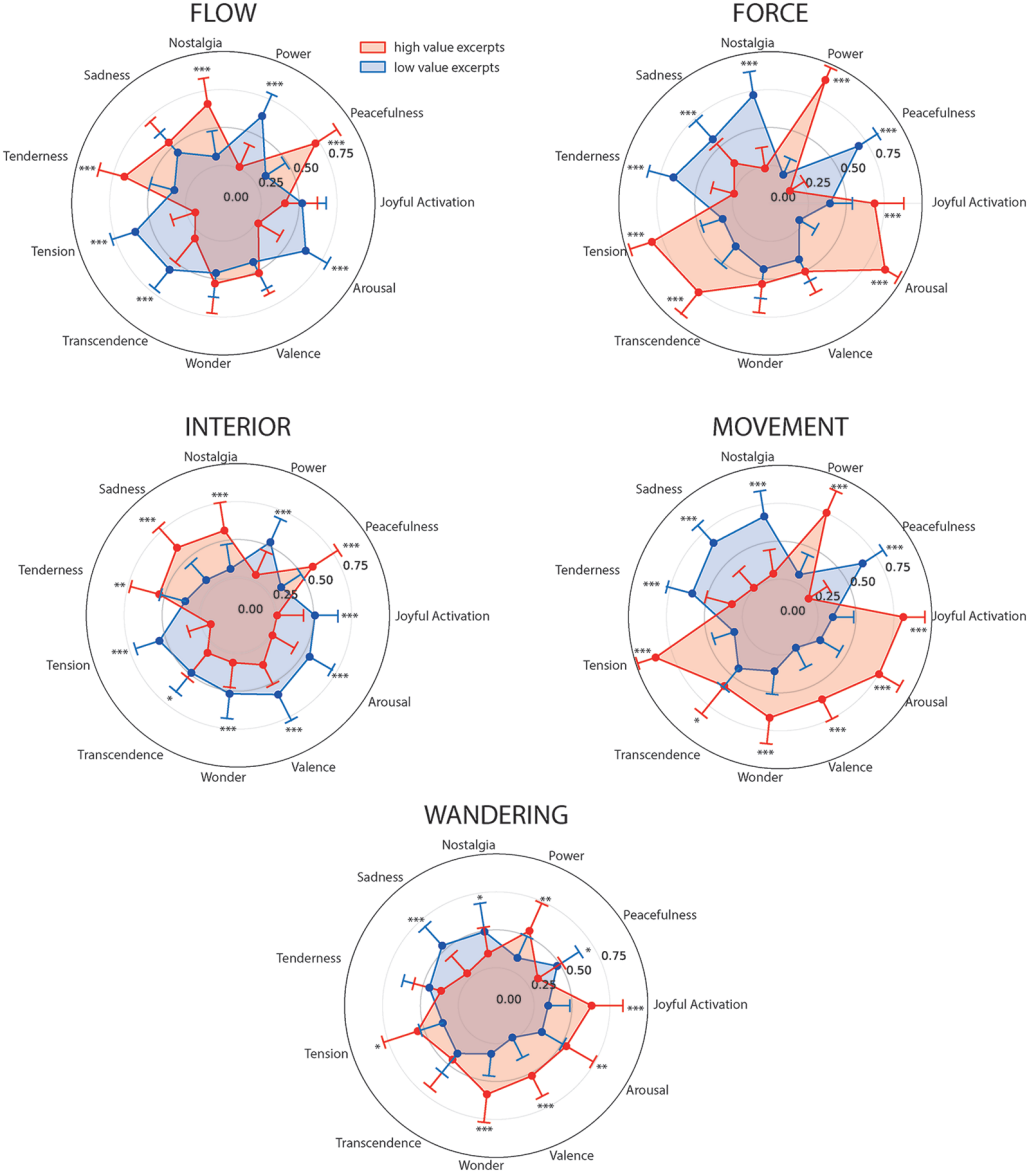
Polar Plot of the Estimated Binary Value of Each Metaphor Based on the Emotional Content of the Musical Excerpts. The Red Shape Represents the Excerpts That Were Rated High for Such Emotional Content. The Blue Shape Represents the Excerpts That Were Rated Low for Such Emotional Content. The Contrasts Compare the Estimated Value of a Specific Metaphor Between the High Value and Low Value Excerpts for Each Affective Term. For Example, a Single Contrast Compares the “Flow” Values Obtained for Excerpts Characterized as High for “Nostalgia” and Low for “Nostalgia.” All Contrasts Are FDR-Corrected [* p<.05 , ** p<.01 , *** p<.001 ].*Note.* Please refer to the online version of the article to view the figure in colour.

**Figure 4.**
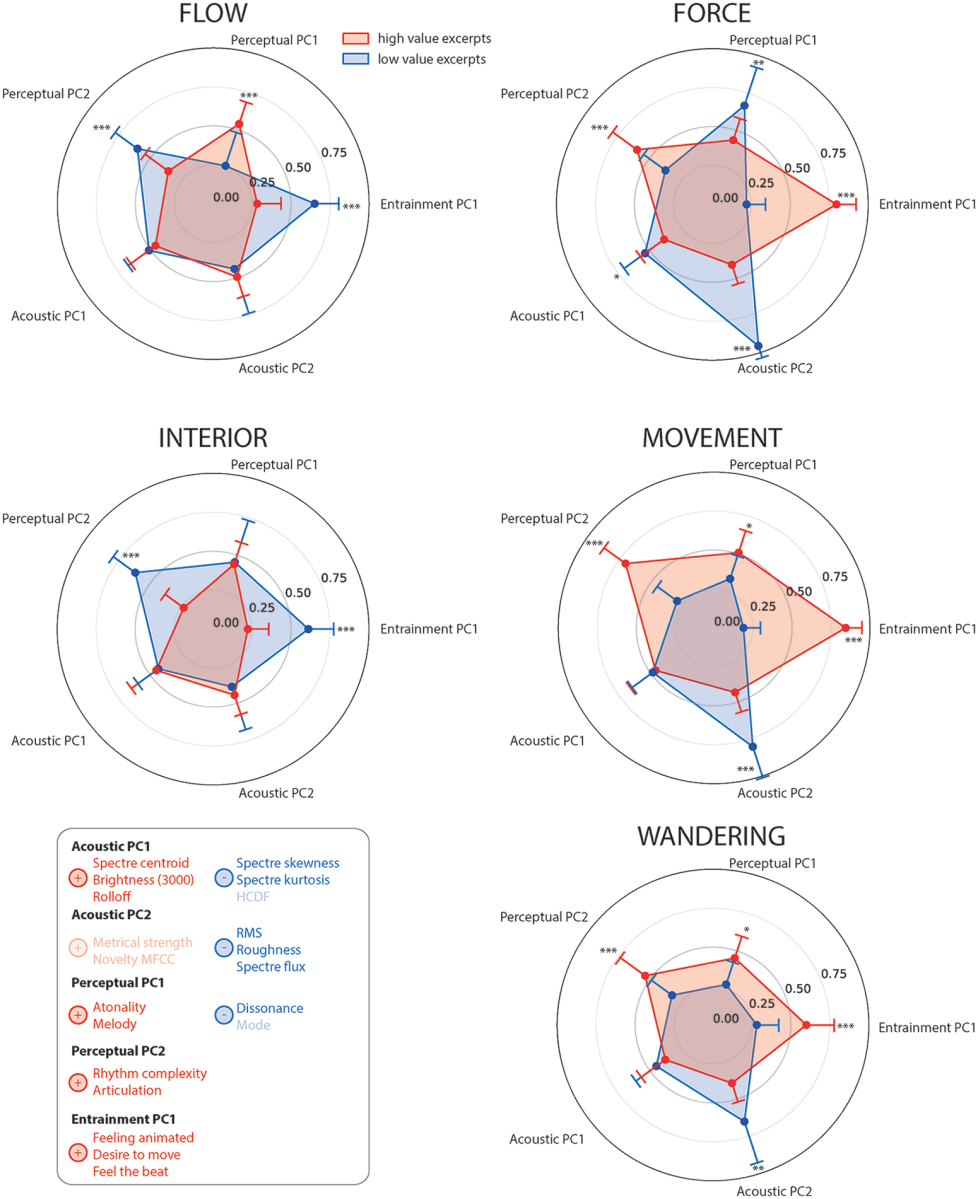
Polar Graph of the Estimated Binary Value of Each Metaphor Based on the Principal Components of the Acoustic and Perceptual Features Associated With the Musical Excerpts. The Red Shape Represents the Excerpts That Were Rated High for Such Descriptors. The Blue Shape Represents the Excerpts That Were Rated Low for Such Descriptors. The Contrasts Compare the Estimated Value of a Specific Metaphor Between the High Value and Low Value Excerpts for Each Component. All Contrasts Are FDR-Corrected [* p<.05 , ** p<.01 , *** p<.001 ]. The Table Summarizes the Principal Components Resulting From the PCA on the Acoustic Feature, the Perceptual Feature, and the Subjective Entrainment Questionnaire. Only the Features With a Weight Superior to 0.5 Are Displayed. Features With a Weight Superior to 0.7 Are as Displayed as Not Faded. Positive Weights Are Shown in Red and Negative in Blue.*Note.* Please refer to the online version of the article to view the figure in colour.

We have supplemented these results by calculating models for each music descriptor. We have labeled each excerpt as high or low for each musical descriptors, based on either the participants’ responses or on the computed acoustical features (Online Supplemental Material C). Models that included an interaction between the metaphors and the descriptor and their main effect always outperformed models with the main effect only (Online Supplemental Material H). We have presented the results in polar diagrams to allow a quick characterization of each metaphor at a glance (Figure 3, for description of the metaphors in terms of emotional content, and Figure 4, for a description of the metaphors in terms of acoustic and perceptual features). Participants reported significantly more “Flow” when excerpts were subjectively associated with (1) more “Peacefulness,” “Nostalgia,” “Tenderness,” and melody and (2) less “Power,” “Tension,” “Transcendence,” “Arousal,” dissonance, subjective entrainment, articulation, and rhythm (Online Supplemental Material I). Participants reported more “Force” for excerpts associated with (1) more “Joyful activation,” “Power,” “Tension,” “Transcendence,” “Arousal,” dissonance, subjective entrainment, articulation, and intensity and (2) less “Peacefulness,” “Nostalgia,” “Sadness,” and “Tenderness.” Participants reported more “Interior” for excerpts associated with (1) more “Peacefulness,” “Nostalgia,” “Sadness,” and “Tenderness” and (2) less “Joyful activation,” “Power,” “Tension,” “Transcendence,” “Wonder,” “Valence,” “Arousal,” subjective entrainment, and articulation. Participants reported more “Movement” when excerpts were associated with (1) more “Joyful activation,” “Power,” “Tension,” “Transcendence,” “Wonder,” “Valence,” “Arousal,” melody, subjective entrainment, articulation, and intensity and (2) less “Peacefulness,” “Nostalgia,” “Sadness,” and “Tenderness.” Finally, participants reported more “Wandering” for excerpts associated with (1) more “Joyful activation,” “Power,” “Tension,” “Wonder,” “Valence,” “Arousal,” articulation, melody, intensity, and subjective entrainment.

### Visualizing relationships

**Figure 5.**
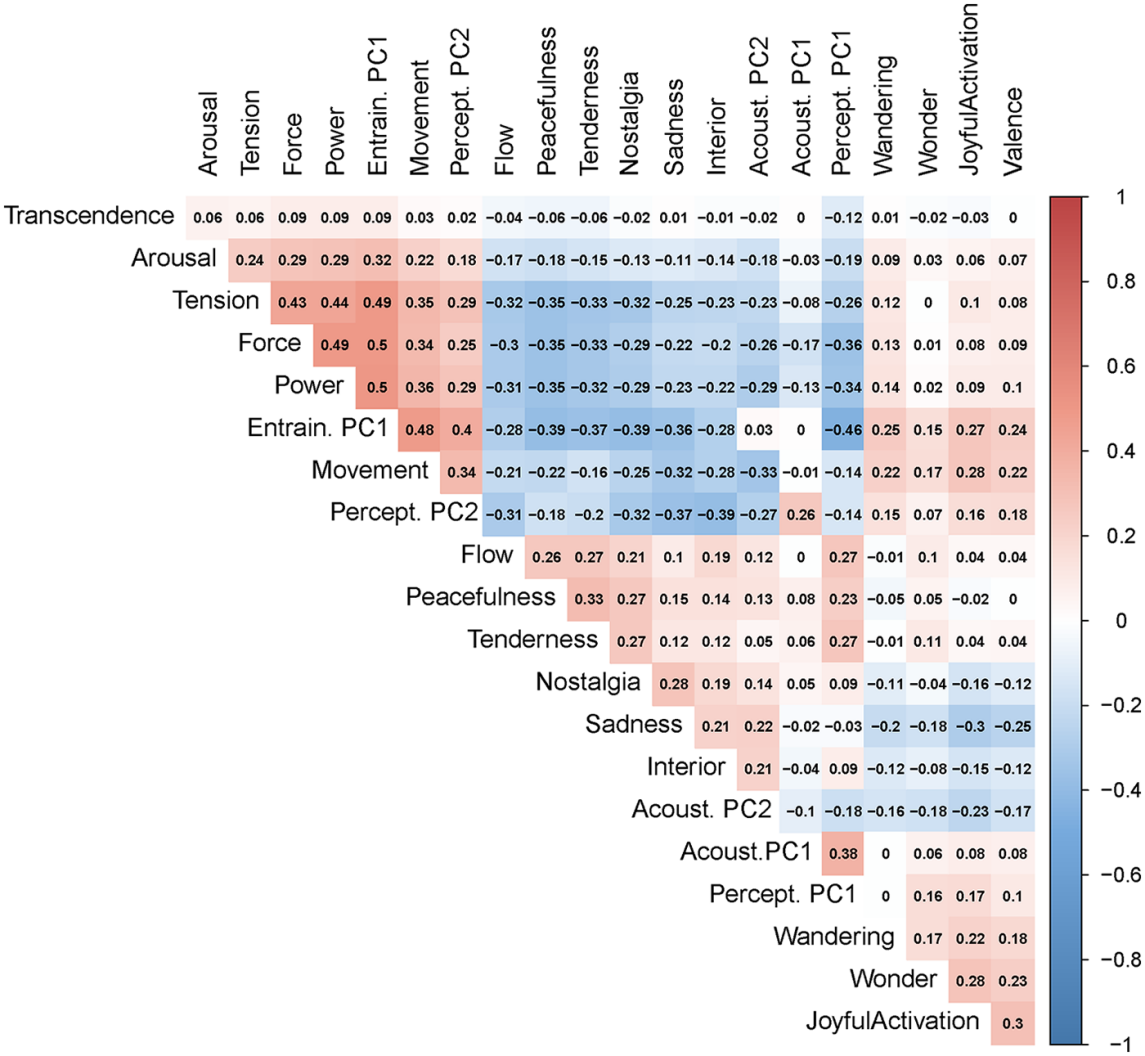
Correlation Table Between Every Item of Every Scale. Correlations were Calculated Based on Permutations of Pairs of Participants.*Note.* Please refer to the online version of the article to view the figure in colour.

**Figure 6.**
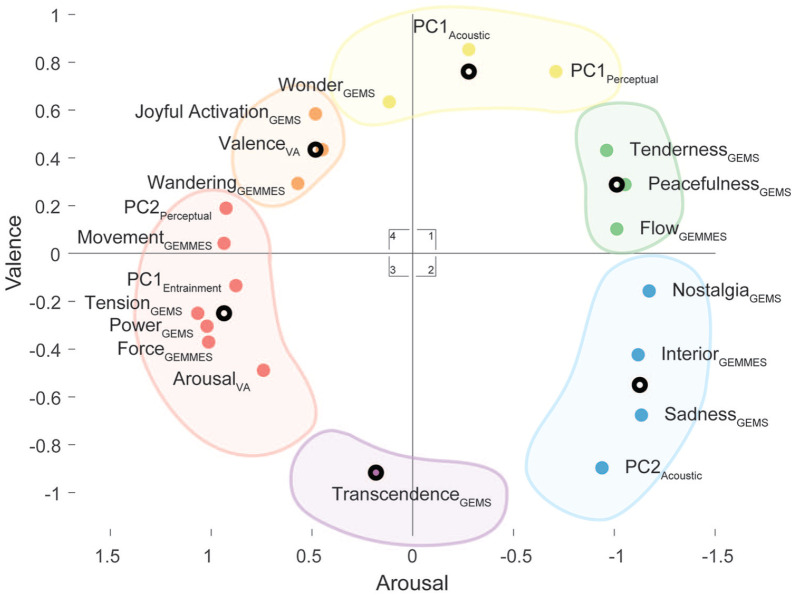
Multidimensional Scaling Based on the Spearman’s Correlations Between Every Item of All Scales and Features. The Six Clusters Are Based on a Clustering k-Means Analysis.*Note.* Please refer to the online version of the article to view the figure in colour.

After several analyses (multiple regression with the best subset selection, multicollinearities)(Online Supplemental Material K), we decided to focus on the correlation matrix. The correlations ranged from negative r=−.46 between the component for subjective entrainment and the first perceptual component to positive r=.5 between “Power” and subjective entrainment (Figure 5). Based on the correlations, we performed a Multidimensional Scaling (MDS) to reduce the dimensionality of our data and graphically represent the correlation between the ratings in two dimensions. We performed a k-means clustering with the city block method on the MDS to group items into clusters. The resulting set of six clusters showed a good compromise between model complexity and accuracy (Figure 6, Online Supplemental Material L). Clockwise around the graph, the first cluster located between quadrants 1 and 4 was associated with “Wonder,” as well as the first components for acoustic and perceptual features. The second cluster located in quadrant 1 featured “Tenderness,” “Peacefulness,” and “Flow.” The third cluster located in quadrant 2 consisted of “Nostalgia,” “Interior,” “Sadness,” and the lack of intensity and roughness. The fourth cluster located in between quadrants 2 and 3 featured “Transcendence” from the GEMS. The fifth cluster located in quadrants 3 and 4 was associated with “Power,” “Tension,” “Arousal,” “Movement,” subjective entrainment, and articulation. Finally, the final cluster in quadrant 4 consisted of “Wandering,” “Valence,” and “Joyful activation.”

## Discussion

The aim of this study was to test the relationships between musical emotions and metaphors, as well as acoustic and perceptual characteristics in connection with listening to Western classical music. For this purpose, we created two different surveys in which the participants had to listen to the same musical excerpts. One focused on the musical metaphors while the other asked participants about musical emotions. We collected the responses from 162 participants and modeled the relationships between emotions and metaphors, as well as acoustic and perceptual features, using GLMMs. Finally, we calculated the correlations and represented graphically such relationships using an MDS approach.

In examining the data collected, we encountered two main results: (1) an accumulated number of zeros compared with other ratings and (2) significant differences between participants with high and low vivid imaginations, but not between musicians and non-musicians. (1) The null ratings can be explained by several reasons; for example, by the design of the scales and the unused elements in each scale (Online Supplemental Material M). We also believe that it may reflect the general ineffability of music, as the participants sometimes “lack the necessary vocabulary to provide accurate verbalizations of their emotional experience” (Zentner & Eerola, 2010; p. 193). This would lead the participants to discard items as they have difficulty connecting the experience with the scales we have provided. (2) The lack of distinction between musicians and non-musicians can be explained by the fact that the meaning is based on physical experience accessible to everyone (Barsalou, 2005). While we do not claim that our participant pool is representative of the entire French-speaking population, the geographical proximity of the population tested favors a homogeneous cultural background for the creation of meaning. However, it seems that a vivid imagination can influence the evaluation of metaphors, at least for metaphors like “Flow,” “Interior,” and “Wandering.” In our study, participants with a vivid imagination rated these metaphors across excerpts higher than those with a less vivid imagination. Even if the human conceptual system is viewed essentially as metaphorical (Lakoff & Johnson, 1980), with figurative language and metaphors occurring roughly every 25–30 words during spoken discourse (Graesser, Mio, & Millis, 1989), some people seem more inclined to use certain metaphors to describe their experience. We believe that the metaphors “Force” and “Movement” have not been influenced as much due to their ubiquitous use in music, which makes them very familiar to everyone (Antovic, 2015; Cumming, 2000). It confirms that metaphors are highly dependent on interindividual variability and context. This is the main disadvantage of using metaphors, for example, in the context of music lessons where students may have difficulty understanding a teacher’s metaphorical language (Persson, 1996).

If we summarize the results of the GLMMs, multiple regression, and MDS, we can safely link musical metaphors to emotions, acoustic and perceptual features. We can distinguish two groups. The first group includes the metaphors “Force,” “Movement,” and “Wandering,” the emotions “Power,” “Tension,” “Joyful activation,” even “Wonder” to some extent, but also “Arousal,” as well as high subjective entrainment, articulation, and intensity. The second group includes the metaphors “Flow” and “Interior,” the emotions “Peacefulness,” “Tenderness,” “Sadness,” and “Nostalgia,” but also low subjective entrainment, more melody, and less dissonance. We attribute this grouping to our ability as humans to perceive music as movement in space. According to Lawrence Barsalou’s (1999) theory of perceptual symbol systems, the human brain can correlate the sequence of musical events with brain maps that have already been generated by other modalities (e.g., vision and taste). For example, several studies reported activation of areas of the brain associated with visual processing during a music-related task (Nakamura et al., 1999; Penhune, Zatorre, & Evans, 1998; Platel et al., 1997; Zatorre, Evans, & Meyer, 1994). It seems that even if the performer cannot be seen, the listener’s brain can process music in terms of the body movements from which the sounds originate (Galati et al., 2008; Gazzola, Aziz-Zadeh, & Keysers, 2006; Hauk, Shtyrov, & Pulvermüller, 2006). Clarke (2005) even suggested that “since sounds in the everyday world specify (among other things) the motional characteristics of their sources, it is inevitable that musical sounds will also specify the fictional movements and gestures of the virtual environment which they conjure up” (p. 74). Musical movement is deeply linked to musical metaphors and emotions as musical meaning is based on embodied cognitive-based kinetic experiences (Aksnes, 2000; Borgo, 2004; Chuck, 2004; Cox, 2001; M. L. Johnson, 1997; Walker, 2000). Hence, metaphors associated with movements such as “Force,” “Movement,” and “Wandering” would of course also relate to movement-related emotions such as “Joyful activation” or “Power,” but also to subjective entrainment as it is connected to bodily movement and dancing. This connection between movement in space and musical meaning is also anchored in our music history, as musicologists have been describing music in terms of movement and energy for thousands of years (Rothfarb, 2002). Today’s musicological writings continue to describe music as a continuous, one-way forward movement through space (Cumming, 2000). Zbikowski (1998) even added: “the concepts of space and motion are extended to music through metaphorical transference as a way to account for certain aspects of our experience of music. These metaphors are not an addition to musical understanding, but are in fact basic to it” (p. 2). By assuming that musical gestures are isomorphic with expressive gestures, the experience of music as movement is seen as an important link between music and emotions by aestheticians (e.g., Hanslick, 1891; Kivy, 1981; Langer, 1953), semioticians (e.g., Lidov, 1999), and music theorists (e.g., Kurth & Ernst, 1991; Spitzer, 2003; Zbikowski, 2002). Despite the common embodied knowledge and the dissemination in musicological writings, the individual remains the final factor in the decision to move or not (or even just imagine motion) when music suggests such a movement, therefore creating a lot of subjectivity in the music and partly explaining the individual differences in the perception of music (Koelsch, 2011).

With MDS, our results were projected onto two dimensions. While the horizontal dimension seemed to relate to arousal (with higher arousal on the left), the vertical axis could be assigned to valence (with positive valence on top). Beyond the musical motion, musical metaphors and emotions therefore also seemed to fit a circumplex model (Russell, 1980). Looking at such a complex musical meaning in terms of two dimensions, valence and arousal, could be useful as it has been recommended as the most efficient and reliable method for collecting and displaying musical emotion data (Vuoskoski & Eerola, 2011), even if some authors disagree and prefer a classification approach (Zentner et al., 2008). While we are not suggesting the solitary use of a dimensional model to describe musical metaphors and emotions, as this would lead to the loss of complex and important nuances, this work supports such a model as a complementary conceptual basis or as a kind of building block for describing the connection between musical metaphors, emotions, acoustics, and perceptual features. We believe that experimenters who want the most differentiated opinion from their participants should ask for free answers. In practice, however, we recommend using at least a combination of GEMS and GEMMES. They should know that the dimensional model is embedded in such scales. In any event, if the researchers want to collect fast and less nuanced data, we recommend using a dimensional model that is a building block of both scales.

There are a few limitations worth mentioning for this study. First, because metaphors are culturally dependent, the sample of participants used in this study reflects only a partial truth. Generalizing these results to other populations will therefore be essential if such results want to be used outside our pool of participants. Second, the procedure used in this work was established to keep metaphors and emotions separate in their evaluation. We used two groups of participants who rated each scale separately to ensure that the musical excerpts, metaphors, and emotions had no exposure effect. Using a repeated measurements design is an alternative to the method we used. This work would benefit from a more direct representation of how each participant rates both metaphors and emotions on the same excerpts. However, we believe that exposure to one would affect the evaluation of the other. Finally, for reasons of ecological validity, this study should include a more diverse variety of musical genres. We chose Western classical music for its absence of lyrics and the possibility to evoke vivid images and metaphors (Band, Quilter, & Miller, 2001; McKinney, Antoni, Kumar, Tims, & McCabe, 1997). It is also the main genre of music explored by music theorists who use metaphors and emotions as common tools in their work. Nowadays, pop/rock is the most widespread music genre (Juslin, Liljeström, Västfjäll, Barradas, & Silva, 2008). We believe that the results of this study should be extended to different genres of music as the metaphors used could change.

After all, we believe music professionals can greatly benefit from the results of this study. Metaphorical and emotional language, as well as meaning in general, have been the cornerstone of several disciplines such as music writing and music education. First, music writings are generally based on conceptual models, which are best explained by metaphors (Zbikowski, 1983). In particular, text painting, although a somewhat less usual compositional technique, points to the basis for metaphorical descriptions of music (Zbikowski, 2008). Erik Satie, a composer, often uses text painting on his music scores to describe an emotion or action, such as “wonder about yourself,” “don’t leave,” “on the tip of the tongue.” This figurative language conveys how the piece should be played and heard and convinces the students to aim for a certain experience and type of performance (Barten, 1998). Second, metaphorical and emotional language plays a central role in music education, especially conceptual metaphors associated with space and gesture (Guck, 1981). It has been shown that they are a particularly effective theoretical tool (Guck, 1994) and educational tool (Woody, 2002). Music teachers uses images and metaphors, which can usually be divided into those that convey mood and emotions (e.g., “sing like you’ve just fallen in love”) and those that depict motion (e.g., “imagine skipping a stone across a lake”) (Woody, 2002). Music educators recommend using this approach (Lindström, Juslin, Bresin, & Williamon, 2003; Woody, 1998), and musicians have shown they are familiar with it (Sheldon, 2004; Woody, 2002). “The metaphor helps the student attain an emergent multidimensional grasp of the music. . . .The metaphor creates an affective state within which the performer can attempt to match the model” (Davidson & Scripp, 1989, p. 95). Obviously, using metaphors in music lessons is a task in itself as metaphors are culturally and linguistically specific. Persson (1996) has identified potential problems for such an approach, including confusion and discouragement on the part of students who struggle to understand a teacher’s metaphorical language (Persson, 1996). In addition, we urge music providers (e.g., Apple Music, Pandora Radio, Spotify, and Google Play Music) to classify their enormous library of songs based on musical emotions and metaphors.

All in all, while our results come from an intrapersonal perspective, in which the listener is only facing the music, the associated effects and implications seem to live on an interpersonal level, from musician to musician, from teacher to student, from musician to listener, and from listener to listener. After all, this work offers scientific and evidence-based reasons for an ancestral intuitive connection between musical experience, metaphors, and emotions. While it proves the solidity of the association of musical metaphors and emotions, it opens up a way to empirically test, measure, and organize such associations in the context of musical experiences.

## Supplemental Material

sj-pdf-1-pom-10.1177_0305735621991235 – Supplemental material for Linking musical metaphors and emotions evoked by the sound of classical music Click here for additional data file. Supplemental material, sj-pdf-1-pom-10.1177_0305735621991235 for Linking musical metaphors and emotions evoked by the sound of classical music by Simon Schaerlaeken, Donald Glowinski and Didier Grandjean in Psychology of Music

## Notes

1. They developed an approach based on two indicators, a marginal and a conditional CR13I1 ( 156242 and $R^{2}$ , respectively), allowing comparability with standard methods, while considering the variance explained by the random effects. $R_{m}^{2}$ is the variance explained by the fixed factors, whereas $R_{c}^{2}$ is the variance explained by the entire model (both fixed and random effects).

## References

[bibr1-0305735621991235] Aksnes H. (2000). Music and its resonating body. In Dansk ärbog for musickforskning [Danish yearbook for music research] (p. 29). Retrieved from http://www.dym.dk/dym_pdf_files/volume_29/volume_29_081_101.pdf

[bibr2-0305735621991235] Aljanaki A. Soleymani M. (2018). A data-driven approach to mid-level perceptual musical feature modeling. arXiv preprint arXiv:1806.04903. Retrieved from https://arxiv.org/pdf/1806.04903.pdf

[bibr3-0305735621991235] Antovic M. (2015). Metaphor in music or metaphor about music: A contribution to the cooperation of cognitive linguistics and cognitive musicology ( Antovic M. , Trans.). In Stanojević M. (Ed.), Metaphors we study: Contemporar (pp. 233–254). Zagreb, Croatia: Srednja Europa.

[bibr4-0305735621991235] Antović M. (2018). From expectation to concepts: Toward multilevel grounding in musical semantics. Cognitive Semiotics, 9, 105–138.

[bibr5-0305735621991235] Band J. P. Quilter S. M. Miller G. M. (2001). The influence of selected music and inductions on mental imagery: Implications for practitioners of Guided Imagery and Music. Journal of the Association for Music and Imagery, 8, 13–33.

[bibr6-0305735621991235] Barsalou L. W. (1999). Perceptual symbol systems. Behavioral and Brain Sciences, 22, 577–660. 10.1017/s0140525x99002149 11301525

[bibr7-0305735621991235] Barsalou L. W. (2005). Abstraction as dynamic interpretation in perceptual symbol systems. Building Object Categories, 30322, 389–431.

[bibr8-0305735621991235] Barten S. S. (1998). Speaking of music: The use of motor-affective metaphors in music instruction. Journal of Aesthetic Education, 32, 89–97.

[bibr9-0305735621991235] Behrens G. A. Green S. B. (1993). The ability to identify emotional content of solo improvisations performed vocally and on three different instruments. Psychology of Music, 21, 20–33.

[bibr10-0305735621991235] Bhattacharya J. Lindsen J. P. (2016). Music for a brighter world: Brightness judgment bias by musical emotion. PLoS ONE, 11(2), Article e0148959. 10.1371/journal.pone.0148959 PMC4749205 26863420

[bibr11-0305735621991235] Bonde L. O. (2007). Music as metaphor and analogy. Nordic Journal of Music Therapy, 16, 73–81.

[bibr12-0305735621991235] Borgo D. (2004). Play of meaning and the meaning of play in jazz, the. Journal of Consciousness Studies, 11, 174–190.

[bibr13-0305735621991235] Casasanto D. Dijkstra K. (2010). Motor action and emotional memory. Cognition, 115, 179–185. 20096831 10.1016/j.cognition.2009.11.002 PMC2830276

[bibr14-0305735621991235] Chuck G. (2004). Musical meaning and cognitive operations of the embodied mind (Unpublished doctoral dissertation). Eastman School of Music, University of Rochester, Rochester, NY.

[bibr15-0305735621991235] Clarke E. F. (2005). Ways of listening: An ecological approach to the perception of musical meaning. Oxford, UK: Oxford University Press.

[bibr16-0305735621991235] Clifton T. (1983). Music as heard. a study in applied phenomenology. London, England: Yale University Press.

[bibr17-0305735621991235] Cooke D. (1959). The language of music. Oxford, UK: Oxford University Press.

[bibr18-0305735621991235] Cooper G. Meyer L. B. (1960). The rhythmic structure of music. Chicago, IL: University of Chicago Press.

[bibr19-0305735621991235] Cox A. (2001). The mimetic hypothesis and embodied musical meaning. Musicae Scientiae, 5, 195–212.

[bibr20-0305735621991235] Cumming N. (2000). The sonic self: Musical subjectivity and signification. Bloomington: Indiana University Press.

[bibr21-0305735621991235] Davidson L. Scripp L. (1989). Education and development in music from a cognitive perspective. In Hargreaves D. J. (Ed.), Children and the arts (pp. 59–86). Milton Keynes, UK: Open University Press.

[bibr22-0305735621991235] Davies S. (1994). Musical meaning and expression. New York, NY: Cornell University Press.

[bibr23-0305735621991235] Dolscheid S. Shayan S. Majid A. Casasanto D. (2013). The thickness of musical pitch: Psychophysical evidence for linguistic relativity. Psychological Science, 24, 613–621. 23538914 10.1177/0956797612457374

[bibr24-0305735621991235] Dowling W. J. Harwood D. L. (1986). Music cognition. Cambridge, MA: Academic Press.

[bibr25-0305735621991235] Eerola T. Vuoskoski J. K. (2011). A comparison of the discrete and dimensional models of emotion in music. Psychology of Music, 39, 18–49.

[bibr26-0305735621991235] Eerola T. Vuoskoski J. K. (2013). A review of music and emotion studies: Approaches, emotion models, and stimuli. Music Perception: An Interdisciplinary Journal, 30, 307–340.

[bibr27-0305735621991235] Eitan Z. Timmers R. (2010). Beethoven the last piano sonata and those who follow crocodiles: Cross-domain mappings of auditory pitch in a musical context. Cognition, 114, 405–422. 20036356 10.1016/j.cognition.2009.10.013

[bibr28-0305735621991235] Ekman P. (1992). An argument for basic emotions. Cognition & Emotion, 6, 169–200.

[bibr29-0305735621991235] Eliard K. (2017). Dynamiques temporelles des emotions exprimees par la musique (Temporal dynamics of emotions evoked by music) (Unpublished doctoral dissertation). University of Geneva, Geneva, Switzerland.

[bibr30-0305735621991235] Elizabeth Crawford L. Margolies S. M. Drake J. T. Murphy M. E . (2006). Affect biases memory of location: Evidence for the spatial representation of affect. Cognition & Emotion, 20, 1153–1169.

[bibr31-0305735621991235] Epstein D. (1995). Shaping time: Music, the brain, and performance. New York, NY: Schirmer Books.

[bibr32-0305735621991235] Fauconnier G. Turner M. (2008). The way we think: Conceptual blending and the mind’s hidden complexities. New York, NY: Basic Books.

[bibr33-0305735621991235] Flowers P. J. Wang C.-h. (2002). Matching verbal description to music excerpt: The use of language by blind and sighted children. Journal of Research in Music Education, 50, 202–214.

[bibr34-0305735621991235] Gabrielsson A. (1995). Expressive intention and performance. In Steinberg R. (Ed.), Music and the mind machine (pp. 35–47). Berlin, Germany: Springer.

[bibr35-0305735621991235] Gabrielsson A. (2001). Emotion perceived and emotion felt: Same or different? Musicae Scientiae, 5, 123–147.

[bibr36-0305735621991235] Gabrielsson A. Juslin P. N. (1996). Emotional expression in music performance: Between the performer’s intention and the listener’s experience. Psychology of Music, 24, 68–91.

[bibr37-0305735621991235] Galati G. Committeri G. Spitoni G. Aprile T. Di Russo F. Pitzalis S. Pizzamiglio L. (2008). A selective representation of the meaning of actions in the auditory mirror system. NeuroImage, 40, 1274–1286. 18276163 10.1016/j.neuroimage.2007.12.044

[bibr38-0305735621991235] Gazzola V. Aziz-Zadeh L. Keysers C. (2006). Empathy and the somatotopic auditory mirror system in humans. Current Biology, 16, 1824–1829. 16979560 10.1016/j.cub.2006.07.072

[bibr39-0305735621991235] Graesser A. C. Mio J. Millis K. (1989). Metaphors in persuasive communication. In Meutsch D. Viehoff R. (Eds.), Comprehension of literary discourse: Results and problems of interdisciplinary approaches (pp. 131–154). Berlin, Germany: Walter de Gruyter.

[bibr40-0305735621991235] Guck M. (1981). Metaphors in musical discourse: The contribution of imagery to analysis (Unpublished doctoral dissertation). University of Michigan, Ann Arbor.

[bibr41-0305735621991235] Guck M. (1994). Analytical fictions. Music Theory Spectrum, 16, 217–230.

[bibr42-0305735621991235] Hanslick E. (1891). The beautiful in music: A contribution to the revisal of musical aesthetics. London, England: H. W. Gray Company.

[bibr43-0305735621991235] Hauk O. Shtyrov Y. Pulvermüller F. (2006). The sound of actions as reflected by mismatch negativity: Rapid activation of cortical sensory–motor networks by sounds associated with finger and tongue movements. European Journal of Neuroscience, 23, 811–821. 10.1111/j.1460-9568.2006.04586.x 16487161

[bibr44-0305735621991235] Johnson M. (1987). The body in the mind: The bodily basis of imagination, reason, and meaning. Chicago, IL: University of Chicago Press.

[bibr45-0305735621991235] Johnson M. L. (1997). Embodied musical meaning. Theory and Practice, 22, 95–102.

[bibr46-0305735621991235] Johnson M. L. Larson S. (2003). “Something in the way she moves”-metaphors of musical motion. Metaphor and Symbol, 18, 63–84.

[bibr47-0305735621991235] Juslin P. N. (2000). Cue utilization in communication of emotion in music performance: Relating performance to perception. Journal of Experimental Psychology: Human Perception and Performance, 26, 1797–1813. 11129375 10.1037//0096-1523.26.6.1797

[bibr48-0305735621991235] Juslin P. N. (2011). Music and emotion: Seven questions, seven answers. In Deliège I. Davidson J. Sloboda J. A. (Eds.), Music and the mind: Essays in honour of John Sloboda (pp. 113–135). Oxford, UK: Oxford University Press.

[bibr49-0305735621991235] Juslin P. N. (2013). From everyday emotions to aesthetic emotions: Towards a unified theory of musical emotions. Physics of Life Reviews, 10, 235–266. 23769678 10.1016/j.plrev.2013.05.008

[bibr50-0305735621991235] Juslin P. N. Laukka P. (2003). Communication of emotions in vocal expression and music performance: Different channels, same code? Psychological Bulletin, 129, 770–814. 12956543 10.1037/0033-2909.129.5.770

[bibr51-0305735621991235] Juslin P. N. Laukka P. (2004). Expression, perception, and induction of musical emotions: A review and a questionnaire study of everyday listening. Journal of New Music Research, 33, 217–238.

[bibr52-0305735621991235] Juslin P. N. Liljestrom S. Laukka P. Vastfjall D. Lundqvist L.-O. (2011). Emotional reactions to music in a nationally representative sample of Swedish adults: Prevalence and causal influences. Musicae Scientiae, 15, 174–207.

[bibr53-0305735621991235] Juslin P. N. Liljeström S. Västfjäll D. Barradas G. Silva A. (2008). An experience sampling study of emotional reactions to music: Listener, music, and situation. Emotion, 8, 668–683. 18837617 10.1037/a0013505

[bibr54-0305735621991235] Juslin P. N. Madison G. (1999). The role of timing patterns in recognition of emotional expression from musical performance. Music Perception: An Interdisciplinary Journal, 17, 197–221.

[bibr55-0305735621991235] Juslin P. N. Västfjäll D. (2008). Emotional responses to music: The need to consider underlying mechanisms. The Behavioral and Brain Sciences, 31, 559–575; discussion 575–621. 18826699 10.1017/S0140525X08005293

[bibr56-0305735621991235] Kivy P. (1981). The corded shell: Reflections on musical expression. Journal of Aesthetics and Art Criticism, 39, 460–462.

[bibr57-0305735621991235] Koelsch S. (2011). Towards a neural basis of processing musical semantics. Physics of Life Reviews, 8, 89–105. 21601541 10.1016/j.plrev.2011.04.004

[bibr58-0305735621991235] Kövecses Z. (2008). Conceptual metaphor theory: Some criticisms and alternative proposals. Annual Review of Cognitive Linguistics, 6, 168–184.

[bibr59-0305735621991235] Krumhansl C. L. (1990). Tonal hierarchies and rare intervals in music cognition. Music Perception: An Interdisciplinary Journal, 7, 309–324.

[bibr60-0305735621991235] Kurth E. Ernst K. (1991). Ernst Kurth: Selected writings (Vol. 2). Cambridge, MA: Cambridge University Press.

[bibr61-0305735621991235] Labbé C. Grandjean D. (2014). Musical emotions predicted by feelings of entrainment. Music Perception: An Interdisciplinary Journal, 32, 170–185.

[bibr62-0305735621991235] Lakoff G. (1993). The contemporary theory of metaphor. In Ortony A. (Ed.), Metaphor and thought (pp. 202–251). Cambridge, MA: Cambridge University Press.

[bibr63-0305735621991235] Lakoff G. Johnson M. (1980). The metaphorical structure of the human conceptual system. Cognitive Science, 4, 195–208.

[bibr64-0305735621991235] Lakoff G. Johnson M. (1999). Philosophy in the flesh (Vol. 4). New York, NY: Basic Books.

[bibr65-0305735621991235] Landau M. J. Meier B. P. Keefer L. A. (2010). A metaphor-enriched social cognition. Psychological Bulletin, 136, 1045–1067. 20822208 10.1037/a0020970

[bibr66-0305735621991235] Langer S. K. (1953). Feeling and form (Vol. 3). London, England: Routledge and Kegan Paul.

[bibr67-0305735621991235] Larson S. (2012). Musical forces: Motion, metaphor, and meaning in music. Bloomington: Indiana University Press.

[bibr68-0305735621991235] Lartillot O. Toiviainen P. (2007, September 10–15). A Matlab toolbox for musical feature extraction from audio. Paper presented at the Proceedings of the 10th International Conference on Digital Audio Effects, Bordeaux, France.

[bibr69-0305735621991235] Levinson J. (1980). What a musical work is. The Journal of Philosophy, 77, 5–28.

[bibr70-0305735621991235] Levitin D. J. Menon V. (2003). Musical structure is processed in language areas of the brain: A possible role for Brodmann Area 47 in temporal coherence. NeuroImage, 20, 2142–2152. 14683718 10.1016/j.neuroimage.2003.08.016

[bibr71-0305735621991235] Levitin D. J. Menon V. (2005). The neural locus of temporal structure and expectancies in music: Evidence from functional neuroimaging at 3 Tesla. Music Perception: An Interdisciplinary Journal, 22, 563–575.

[bibr72-0305735621991235] Lidov D. (1999). Elements of semiotics. London, England: Macmillan.

[bibr73-0305735621991235] Lindström E. Juslin P. N. Bresin R. Williamon A. (2003). Expressivity comes from within your soul: A questionnaire study of music students’ perspectives on expressivity. Research Studies in Music Education, 20, 23–47.

[bibr74-0305735621991235] Marks D. F. (1973). Visual imagery differences in the recall of pictures. British Journal of Psychology, 64, 17–24. 4742442 10.1111/j.2044-8295.1973.tb01322.x

[bibr75-0305735621991235] McKinney C. H. Antoni M. H. Kumar M. Tims F. C. McCabe P. M. (1997). Effects of guided imagery and music (GIM) therapy on mood and cortisol in healthy adults. Health Psychology, 16, 390–400. 9237092 10.1037//0278-6133.16.4.390

[bibr76-0305735621991235] Nakagawa S. Schielzeth H. (2013). A general and simple method for obtaining R2 from generalized linear mixed-effects models. Methods in Ecology and Evolution, 4, 133–142.

[bibr77-0305735621991235] Nakamura S. Sadato N. Oohashi T. Nishina E. Fuwamoto Y. Yonekura Y. (1999). Analysis of music–brain interaction with simultaneous measurement of regional cerebral blood flow and electroencephalogram beta rhythm in human subjects. Neuroscience Letters, 275, 222–226. 10580715 10.1016/s0304-3940(99)00766-1

[bibr78-0305735621991235] Pannese A. Rappaz M. A. Grandjean D. (2016). Metaphor and music emotion: Ancient views and future directions. Consciousness and Cognition, 44, 61–71. 27362475 10.1016/j.concog.2016.06.015

[bibr79-0305735621991235] Patel A. D. (2003). Language, music, syntax and the brain. Nature Neuroscience, 6, 674–681. 12830158 10.1038/nn1082

[bibr80-0305735621991235] Peltola H.-R. Saresma T. (2014). Spatial and bodily metaphors in narrating the experience of listening to sad music. Musicae Scientiae, 18, 292–306.

[bibr81-0305735621991235] Penhune V. B. Zatorre R. J. Evans A. C. (1998). Cerebellar contributions to motor timing: A pet study of auditory and visual rhythm reproduction. Journal of Cognitive Neuroscience, 10, 752–765. 9831742 10.1162/089892998563149

[bibr82-0305735621991235] Perlovsky L. I. (2007). Neural dynamic logic of consciousness: The knowledge instinct. In Perlovsky L. I. Kozma R. (Eds.), Neurodynamics of cognition and consciousness (pp. 73–108). Berlin, Germany: Springer.

[bibr83-0305735621991235] Persson R. (1996). Brilliant performers as teachers: A case study of commonsense teaching in a conservatoire setting. International Journal of Music Education, 28, 25–36.

[bibr84-0305735621991235] Piaget J. Inhelder B. (1969). The psychology of the child. New York, NY: Basic Books.

[bibr85-0305735621991235] Platel H. Price C. Baron J.-C. Wise R. Lambert J. Frackowiak R. Eustache F. (1997). The structural components of music perception. a functional anatomical study. Brain: A Journal of Neurology, 120, 229–243. 9117371 10.1093/brain/120.2.229

[bibr86-0305735621991235] Rothfarb L. (2002). Energetics. In Christensen T. (Ed.), The Cambridge history of western music theory (pp. 927–955). Cambridge, MA: Cambridge University Press.

[bibr87-0305735621991235] Russell J. A. (1980). A circumplex model of affect. Journal of Personality and Social Psychology, 39, 1161–1178. 10.1037//0022-3514.79.2.286 10948981

[bibr88-0305735621991235] Schaerlaeken S. Glowinski D. Rappaz M.-A. Grandjean D. (2019). “Hearing music as. . .”: Metaphors evoked by the sound of classical music. Psychomusicology: Music, Mind, and Brain, 29, 100–116.

[bibr89-0305735621991235] Scherer K. R. Zentner M. R. (2001). Emotional effects of music: Production rules. Music and Emotion: Theory and Research, 16, 361–392.

[bibr90-0305735621991235] Scherer K. R. Zentner M. R. Schacht A. (2001). Emotional states generated by music: An exploratory study of music experts. Musicae Scientiae, 5, 149–171.

[bibr91-0305735621991235] Scruton R. (1999). The aesthetics of music. Oxford, UK: Oxford University Press.

[bibr92-0305735621991235] Sheldon D. A. (2004). Listeners’ identification of musical expression through figurative language and musical terminology. Journal of Research in Music Education, 52, 357–368.

[bibr93-0305735621991235] Spitzer M. (2003). The metaphor of musical space. Musicae Scientiae, 7, 101–120.

[bibr94-0305735621991235] Stone R. M. (1981). Toward a Kpelle conceptualization of music performance. The Journal of American Folklore, 94, 188–206.

[bibr95-0305735621991235] Vuoskoski J. K. Eerola T. (2011). Measuring music-induced emotion: A comparison of emotion models, personality biases, and intensity of experiences. Musicae Scientiae, 15, 159–173.

[bibr96-0305735621991235] Walker M. E. (2000). Movement and metaphor: Towards an embodied theory of music cognition and hermeneutics. Bulletin of the Council for Research in Music Education, 145, 27–42.

[bibr97-0305735621991235] Woody R. H. (1998). Music in the education of young adolescents. Middle School Journal, 29, 41–47.

[bibr98-0305735621991235] Woody R. H. (2002). Emotion, imagery and metaphor in the acquisition of musical performance skill. Music Education Research, 4, 213–224.

[bibr99-0305735621991235] Zatorre R. J. Evans A. C. Meyer E. (1994). Neural mechanisms underlying melodic perception and memory for pitch. Journal of Neuroscience, 14, 1908–1919. 8158246 10.1523/JNEUROSCI.14-04-01908.1994 PMC6577137

[bibr100-0305735621991235] Zbikowski L. M. (1983). Conceptual models and cross-domain mapping: New perspectives on theories of music and hierarchy. Journal of Music Theory, 41, 193–225.

[bibr101-0305735621991235] Zbikowski L. M. (1998). Metaphor and music theory: Reflections from cognitive science. Music Theory Online, 4, 1–8.

[bibr102-0305735621991235] Zbikowski L. M. (2002). Conceptualizing music: Cognitive structure, theory, and analysis. Oxford, UK: Oxford University Press.

[bibr103-0305735621991235] Zbikowski L. M. (2008). Metaphor and music. In Gibbs, Jr. R. W. (Ed.), The Cambridge handbook of metaphor and thought (pp. 502–524). Cambridge, MA: Cambridge University Press.

[bibr104-0305735621991235] Zentner M. Eerola T. (2010). Self-report measures and models. In Juslin P. N. Sloboda J. A. (Eds.), Handbook of music and emotion (pp. 187–221). Oxford, UK: Oxford University Press.

[bibr105-0305735621991235] Zentner M. Grandjean D. Scherer K. R. (2008). Emotions evoked by the sound of music: Characterization, classification, and measurement. Emotion, 8, 494–521. 18729581 10.1037/1528-3542.8.4.494

