# Supplementary material for: Linking musical metaphors and emotions evoked by the sound of classical music
Source: Psychol Music. 2021 Mar 24;50(1):245–64. doi: 10.1177/0305735621991235 (PMC8750148; doi:10.1177/0305735621991235)
Supplement: sj-pdf-1-pom-10.1177_0305735621991235 – Supplemental material for Linking musical metaphors and emotions evoked by the sound of classical music [file sj-pdf-1-pom-10.1177_0305735621991235.pdf]

Online Supplementary Material A

Multiple conceptual and experimental theories have tried to link metaphors and emotions, or at least included both entities in their framework. The BRECVEMA, one of the most influential models trying to explain the link between music and emotion, places visual imagery as one of the eight mechanisms to induce emotions (Juslin, 2013). Visual imagery is a conceptualization of the musical structures through a "metaphorical non-verbal mapping between the music and so-called image schemata grounded in bodily experience" (Juslin and Västfjäll (2008), p566)). These inner images can represent an emotional character through a metaphorical mapping of the musical structure (Osborne, 1981), especially with certain acoustical features, such as pitch, dynamics, predictability in melodic, harmonic and rhythmic elements. By manipulating acoustic features, researchers have shown that most musical parameters significantly affect several dimensions of motion imagery. For example, a crescendo will be seen as going upwards. Visual imagery, however, is depending a lot on individual differences and is a very conscious process with a strong volitional influence (Juslin, 2013). A smaller proportion of the population report using such mechanism to elicit emotions (Juslin et al., 2011). More generally, individuals exhibit differences in how vivid they imagination is (Marks, 1973). Finally, visual imagery is also more difficult to manipulate that other processes (such as expectancy) (Juslin, Harmat, & Eerola, 2014). Another theory comes from semiotics, the study of signs. Music can, in a sense, be represented as a kind of sign, a stimulus pattern that has meaning (theory created by Charles S. Pierce in the late 19th century, although not on music per se). If we admit that music is a kind of sign, we must differentiate between the three different types of signs explaining how meaning is attached to the pattern: the icon, the index, and the symbol. These three signs can be applied to music as what Koelsch refers to as extra-musical meaning (Koelsch, 2011). The first type of sign attached to musical meaning is the icon. Iconic musical meaning emerges from the static likeness between musical patterns (and their interpretation) and sounds made of objects, as well as the qualities of objects and even abstract concepts. For example, a musical excerpt may sound 'like a bird', or a piece of music like Louis Moreau Gottschalk's "The banjo" might give an iconic representation of the sound produced by a banjo without using the actual instrument (Zbikowski, 2010). While Charles S. Pierce regarded music mainly as an iconic sign, there are also many features of musical expression that can be indexical and even symbolic (Monelle, 1991). A musical index refers to a sound pattern that indicates the presence of a psychological state of an individual, such as the presence of an emotion or an intention. An example would be "this piece sounds happy". The last type of sign, the symbolic meaning is not studied in this paper, since it depicts a stable habitual convention-based relationship with its object and is associated in general with words, and in the case of music, with national anthems. The next theory concerns conceptual blending. The fathers of this theory, Fauconnier and Turner (2008), propose that a conjunction of conceptual blending and cognitive metaphor offers useful insights for analyzing musical emotions (Fauconnier & Turner, 2008). Conceptual blending is the creation of a novel, emergent structure, occurring in the blended space, from the mapping of two separate input mental spaces, containing each conceptual packet. This mapping is motived by another generic space, hosting preconceptual and often spatial topologies common to the two inputs. Using conceptual blending, Spitzer (2018) managed to explain musical anger, in both Vivaldi and Haydn pieces, as a blending of one reference space containing the emotion scripts (e.g. how anger is supposed to unfold) with the presentation space containing the musical script, performance, and gesture (Spitzer, 2018). Both of them are based on the generic space containing the musical persona, the music's virtual "body". The last theory, useful from our perspective to understand the complex relationships between metaphor, music, and emotion, is based on a hierarchical system of six contextual constraints to build meaning (Antovic, 2018). These proposed six levels are physiological reactions, image-schematic structures, connotational, conceptual, cultural context, and finally personal

experiences. Metaphors based on the grounded image schemata appears at level two while level three ascribe emotional qualities to the music. In addition, emotional connotation can be grounded in the image-schematic level two, when, for instance, an ascending sequence of notes, tapping into the VERTICALITY schema, is interpreted as "majestic" (Cooke, 1959). As the fifth level, culture plays an important role in shaping the musical meaning. Western European music for example features repetitively themes such as the hunt, the military, and the pastoral, that can be related to literature, social history, and the fine arts (Monelle, 2006). Finally, the last level relates to individual differences and make music meaning such a personal experience.

The use of embodiment as a main framework to explain the link between emotions and metaphors in the context of musical meaning has already been explored in two main aforementioned theories: the conceptual blending (Fauconnier & Turner, 2008) and the multilevel grounding in musical semantics (Antovic, 2018). The conceptual blending is the mapping of two input spaces, based on a generic space, that creates a blend from which additional meaning emerges. In the case of music, these input spaces have been associated on one hand with musical features (e.g. notes, performance gestures, musical structure) and on the other hand with different aspects of either an affective event (Spitzer, 2018) or an object/concept (e.g. path) (Antovic, 2015). The act of explaining one domain (or input space) in terms of another is founding ground of the Conceptual Metaphor Theory (CMT, Lakoff & Johnson, 1980}). In such theory, a metaphor is defined as a cross-domain mapping in the conceptual system (Lakoff, 1993). These mappings are based on dynamic cognitive constructs resulting from the embodiment of physical experiences, called image schemata. While not all conceptual metaphors are necessarily grounded in bodily experiences, most might (Goschler, 2005). Similarly, these embodied experiences could support cross-domain mappings with affective domains. Juslin, for the BRECVEMA system, defined emotions themselves as "embodied phenomena that serve to guide action" (Juslin, 2013). Consequently, both metaphors and emotions could share similar embodied cognitive structures linking them in a similar fashion to the music. In the second theory, the multilevel grounding in musical semantics, the elaboration of musical meaning is based on a hierarchical process covering successively physiological, image schematic, connotational, conceptual, elaborated cultural, and individual aspects (Antovic, 2018). The third level, the connotation level, refers to emotions associated with images schemata. For example, an ascending musical sequence (VERTICALITY) could be perceived as "majestic" (Cooke, 1959). The fourth level, the conceptual level, is entirely referential or extramusical, connecting music to the experience of the world and creating conceptual metaphors. Both levels are based upon the image schematic level, referring to the embodied experiences. Consequently, looking at both theories, we suggest that embodied cognition might be at the basis of the link between musical metaphors and emotions in the context of musical meaning.

In this work, we have demonstrated that musical meaning in the form of metaphors and emotions can be extracted from Western classical excerpts. Furthermore, we highlighted that both musical metaphors and emotions seem to be connected in certain ways. Going back to the different theories introduced in this Supplementary Material, we want to link the results presented here to what other researchers have theorized. First, concerning the BRECVEMA, visual imagery, one of the eight mechanisms eliciting emotions when listening to music, seems to be a subset of all the cross-domain mappings possible (Juslin, 2013). Indeed, its visual nature is somewhat limiting the diversity of possible metaphors elicited by music. Moreover, the mechanism appears at the same time as music listening, while the metaphorical meaning of a piece can be explored after listening. Despite these limitations, we have shown that metaphors are associated with different emotions, as it is assumed by the BRECVEMA model. The directionality and timing of such interaction could unfortunately not be observed in this study and remains an important point to clarify in future research. Second, as hinted by

Koelsch, listeners were also able to decode the extra-musical meaning associated with our excerpts, both the iconic and indexal signs (Koelsch, 2011). The coherence in their answers points at the ability of our participants to pick up on the same characteristics in music, such as acoustic and perceptual cues. Lastly, the last two theories introduced in this paper, the conceptual blending and the hierarchical system of six contextual constraints, states the metaphors as a primary building block to explain meaning and musical emotions. Once again, the order of the mechanisms by which the creation of meaning occurs could not be studied in this assessment. Future studies could focus on the temporality of musical emotions and metaphors, as they most likely evolve over time, similar to emotional prosody (Pell & Kotz, 2011, Schaerlaeken e al., 2018).

References Online Supplementary Material A

Juslin, P. N. (2013). From Everyday Emotions to Aesthetic Emotions: Towards a Unified Theory of Musical Emotions. *Physics of Life Reviews*, *10*(3), 235–266

Juslin, P. N., & Västfjäll, D. (2008, oct). Emotional responses to music: the need to consider underlying mechanisms. *The Behavioral and brain sciences*, *31*(5), 559–75; discussion 575–621.

Osborne, J. W. (1981) The mapping of thoughts, emotions, sensations, and images as responses to music, *Journal of Mental Imagery*. Brandon House., 5(1), pp. 133–136.

Juslin, P. N., Liljestrom, S., Laukka, P., Vastfjall, D., & Lundqvist, L.-O. (2011). Emotional reactions to music in a nationally representative sample of Swedish adults: Prevalence and causal influences. *Musicae Scientiae*, *15*(2), 174–207.

Marks, D. F. (1973). Visual imagery dierences in the recall of pictures. *British journal of Psychology*, *64*(1), 17–24

Juslin, P. N., Harmat, L., & Eerola, T. (2014). What makes music emotionally significant? exploring the underlying mechanisms. *Psychology of Music*, *42*(4), 599–623.

Koelsch, S. (2011). Towards a neural basis of processing musical semantics. *Physics of life reviews*, *8*(2), 89–105.

Zbikowski, L. M. (2010). Music, emotion, analysis. *Music Analysis*, *29*(1-3), 37–60.

Monelle, R. (1991). Music and the peircean trichotomies. *International Review of the Aesthetics and Sociology of Music*, 99–108.

Fauconnier, G., & Turner, M. (2008). *The way we think: Conceptual blending and the mind's hidden complexities*. Basic Books.

Spitzer, M. (2018). Conceptual blending and musical emotion. *Musicae Scientiae*, *22*(1), 24–37.

Antovic, M. (2018). From expectation to concepts: Toward multilevel grounding in musical semantics. *Cognitive Semiotics*, *9*(2), 105–138.

Monelle, R. (2006). *The musical topic: hunt, military and pastoral*. Indiana University Press.

Antovic, M. (2015). Metaphor in music or metaphor about music: A contribution to the cooperation of cognitive linguistics and cognitive musicology. *AntoviÊ, M.(2014): Metaphor about music or metaphor in music: A contribution to the cooperation of cognitive linguistics and cognitive musicology [Metafora o muzici ili metafora u muzici: jedan prilog za*

saradnju kognitivne lingvistike i kognitivne muzikologije]. In: Metaphors We Study: Contemporar

Lako, G., & Johnson, M. (1980). The Metaphorical Structure of the Human Conceptual System. *Cognitive Science*, *4*(2), 195–208.

Lako, G. (1993). The contemporary theory of metaphor. In A. Ortony (Ed.), *Metaphor and thought* (pp. 202–251). Cambridge University Press.

Goschler, J. (2005). Embodiment and body metaphors. *Metaphorik. de*, *9*(2005), 33–52.

Pell, M. D., & Kotz, S. A. (2011). On the time course of vocal emotion recognition. *PLoS One*, *6*(11), e27256.

Schaerlaeken, S., & Grandjean, D. (2018). Unfolding and dynamics of affect bursts decoding in humans. *PloS one*, *13*(10), e0206216.

Online Supplementary Material B

Music excerpts were selected based on a pilot study with 20 participants (9 females, age M = 26.2, SD = 6.8) and with the help of a music expert. The list of excerpts presented in the pilot study was drawn from our previous experiment (Schaerlaeken et al., 2019). All excerpts were pieces of western classical music, from different periods of time and different genres. We selected two excerpts per emotion described by the Geneva Emotion Musical Scale (GEMS (Zentner et al., 2008)). For each of the 9 emotions, we selected final excerpts following two criteria: 1) high ratings in the pilot study for a specific emotion, preferably with high discriminability when possible, 2) consensus with the description of the excerpt as assessed by a group of musicians including a professor from the Haute Ecole de Music of Geneva. Since both "Wonder" and "Transcendence" scales were not well recognized in the pilot study, we used four excerpts from another study based on the results obtained for such emotions to complete our list (Eliard et al., 2017). The final list contained 18 excerpts (Table A). During the study, these excerpts were also rated for how much participants knew them. If the participants did not know them, this would ensure that the metaphors and emotions collected on these excerpts are not a result of episodic memory irrelevant to the music itself.

Musical excerpts

Excerpt name	Style	Year	Known
György Kurtág, 12 Microludes for String Quartet, Op. 13 "Hommage à András Mihály": V. Lontano, calmo, appena sentito	Baroque	1978	0.61
Mahler, Gustav. Symphony No.1 in D major. First Movement Bars 1-34	Romantism	1887	0.97
Marais: La Rêveuse, Quatrième Livre	Baroque	1717	1.12
Bach J.-S., The Well-Tempered Clavier, Book II: Prelude No. 19 in A Major, BWV 888	Baroque	1744	1.4
Bach J.-S., 'Goldberg' Variations, BWV 988 (Aria With Divers Variations): Variation 13 a 2 Clav.	Baroque	1740	1.52
Dietrich Buxtehude, Sonata In G, BuxWV 271: Allegro	Baroque	1700	1.66
Vivaldi, Concerto for Flute and Strings in D Major, Op. 10, No. 3, R. 428 - "Il gardellino": 2. Cantabile	Baroque	1728	1.68
Bach J.-S., Brandenburg Concerto No.1 in F, BWV 1046: II. Adagio	Baroque	1721	1.72
Bach J.-S., Passacaglia in C Minor, BWV 582	Baroque	1720	1.76
Francesco Geminiani, Concerto Grosso in C minor, Op. 2, No. 2: III. Adagio	Baroque	1732	1.76
Stravinsky, Le Sacre du Printemps: VIIb. Dance of the Earth	Modern	1913	1.94
Beethoven, Ludwig Van. Violin Concerto in D major, op.61. Rondo. Allegro, Bars 8-101	Baroque	1806	2.21
Bach J.-S., Das Wohltemperierte Klavier, Book 1: Prelude in C Minor, BWV 847	Baroque	1722	2.42
Bach J.-S., Partita No. III, BWV 1006: I. Preludio	Baroque	1720	2.52
Liszt, Liebestraum No. 3 in A Flat, S. 541 No. 3: Notturno III: O Lieb, so lang du lieben kannst (Poco allegro, Con affetto)	Romantism	1850	3.09
Bruch, Max. Violin Concerto No.1 in G minor, op.26. Finale: Allegro energico, Bars 1-115	Romantism	1866	4.12
Tchaïkovski, Piotr Ilitch. Swan Lake, ballet, op.20. Act two Bars 1-51	Romantism	1875	4.6
John Williams, The Imperial March	Modern	1980	7.07

Table A. **Western classical excerpts presented in the experiment with style and year of composition associated**. Excerpts are sorted by how much they are known by the participants (0 = not at all, 4 = neither known nor unknown, 8 = fully known).

Reference for Supplementary Material B

Schaerlaeken, S., Glowinski, D., Rappaz, M.-A., & Grandjean, D. (2019). “hearing music as...”: Metaphors evoked by the sound of classical music. *Psychomusicology: Music, Mind, and Brain*, *29*(2-3), 100.

Zentner, M., Grandjean, D., & Scherer, K. R. (2008, aug). Emotions evoked by the sound of music: characterization, classification, and measurement. *Emotion (Washington, D.C.)*, *8*(4), 494–521.

Eliard, K. (2017). *Dynamiques temporelles des emotions exprimees par la musique* (Unpublished doctoral dissertation). University of Geneva.

Online Supplementary Material C

The descriptors linked to acoustical and perceptual features were principal components (PC) from three PCA computed on the acoustic features, the perceptual features, and the entrainment questionnaire, respectively. The first PCA resulted in two components for the acoustical features that explained cumulatively 48.3% of the variance (respectively 27.05% and 21.27%). The first PC was positively associated with the spectral centroid and brightness. The second PC was negatively associated with the intensity of the signal (RMS) and the roughness. The second PCA, based on the perceptual features, resulted also in two components that encapsulated 65.3% of the variance (respectively 39.7% and 25.6%). The first PC was positively associated with melody and negatively with dissonance. The second PC was positively associated with rhythm and articulation. Finally, the last PCA integrated the entrainment questionnaire into one single component that explained 85.5% of the variance and was associated positively to feeling animated, wanting to move, and feeling the beat.

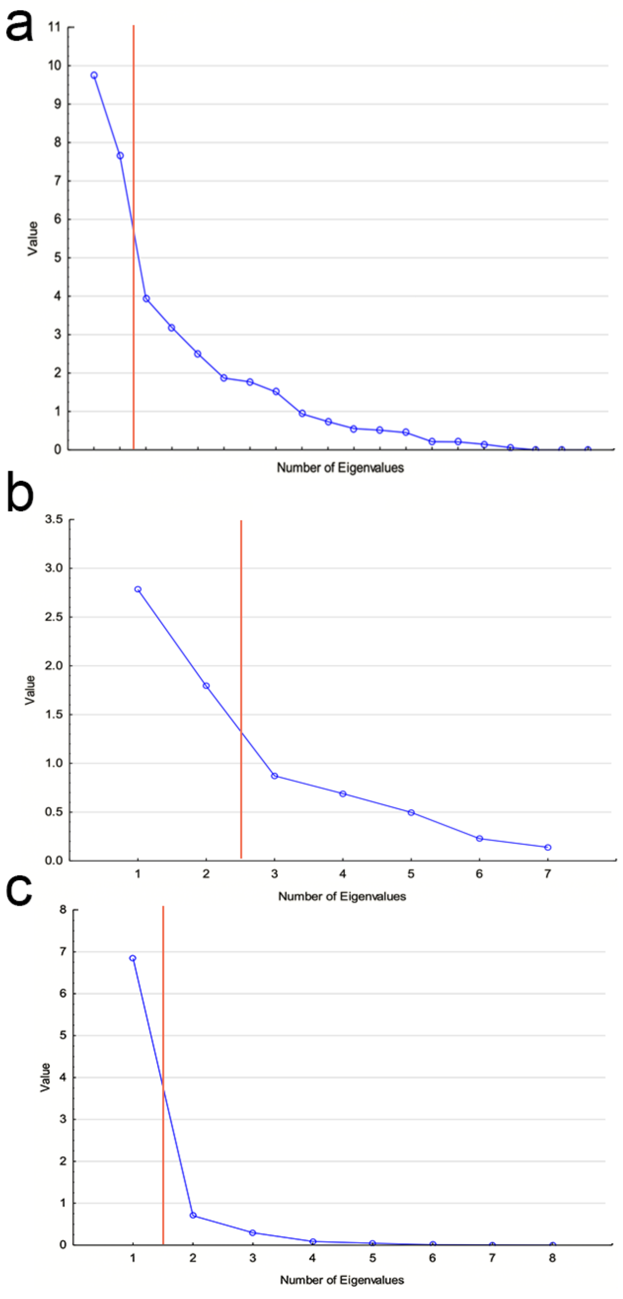

Figures A, B, C. **Scree plot of the eigenvalues for each PCA.** A, acoustic features; B. perceptual features; C. entrainment questionnaire. The red bar show the cutoff selected.

A

Acoustic Features		
Feature	PC1	PC2
SPECTRE_CENTROID	0.940678	0.035345
BRIGHTNESS_3000	0.938519	-0.251984
ROLLOFF	0.847769	0.103166
BRIGHTNESS_1500	0.819710	-0.406413
SPECTRE_FLATNESS	0.782766	0.290597
SPECTRE_SPREAD	0.764693	0.364758
SPECTRE_ENTROPIE	0.749629	-0.490503
BRIGHTNESS_1000	0.736531	-0.430271
FLUX_DU_CEPSTRE	0.702716	0.355707
ZEROCROSS	0.654469	-0.557466
CEPSTRE_MEAN	0.570553	0.135845
TEMPO	0.371219	-0.170255
FLUX_DU_SPECTRE	0.194242	-0.896754
CEPSTRE_MAX	0.156461	-0.171714
TEMPO_CHANGE	0.151476	-0.085444
NOVELTY_METRIC	0.140619	0.438252
NOVELTY_CHROMAGRAM_1	0.103249	0.325284
METRICAL_CENTROID	0.099184	0.102914
ROUGHNESS	0.090817	-0.898217
KEYCLARITY	0.088799	-0.067845
NOVELTY_KEYSTRENGTH_2	0.058246	0.197251
NOVELTY_MFCC	0.021970	0.510698
NOVELTY_KEYSTRENGTH_1	0.019390	-0.103011
NOVELTY_AC_WAVEFORM	0.007971	-0.007376
NOVELTY_CHROMAGRAM_2	0.002343	0.064032
RMS	-0.044912	-0.903057
CEPSTRE_CENTROID	-0.064186	0.240669
NOVELTY_SPECTRE	-0.065941	0.183973
MODE	-0.095622	0.050468
EVENTDENSITY	-0.105223	-0.697273
NOVELTY_CEPSTRE	-0.155961	-0.384054
METRICAL_STRENGTH	-0.407177	0.551147
PULSECLARITY	-0.422190	-0.323303
HCDF	-0.614583	-0.224264
SPECTRE_KURTOSIS	-0.854768	0.082203
SPECTRE_SKEWNESS	-0.912317	0.170225

B

Entrainment Questionnaire	
Items	PC1
Animated	0.993929
Move	0.978406
Beat	0.969453
Entrained	0.968070
Agitated	0.937429
Resonate	0.900178
Physiology	0.837516
Dance	0.794531

C

Perceptual Features		
Feature	PC1	PC2
Melody	0.854216	0.071005
Atonality	0.797815	-0.114160
Rhythm Stabilit	0.324811	0.631492
Rhythm complex	-0.224835	0.799236
Articulation	-0.288898	0.760891
Mode	-0.521771	-0.398711
Dissonnance	-0.895100	0.317213

Tables A, B, C. **Principal component loadings for each PCA**. Only the features with a weight superior to 0.5 are displayed in color. Features with a weight superior to 0.7 a displayed not faded. Positive weights are represented in read and negative in blue.

Online Supplementary Material D

At first glance, the distribution of ratings for both the GEMMES and GEMS scales did not follow a normal distribution. Both were zero-inflated distributions due to the tendency of our participants to associate each excerpt with only a part of the items proposed, leaving the rest at the minimum value. This had two consequences: first, statistical comparison of the raw ratings required non-parametric testing (permutation testing and Spearmann correlation). Second, we artificially created a binomial distribution by characterizing each scale of each trial as "1" or "0". A scale was set to "1" if the corresponding raw rating was superior or equal to the middle of the rating scale (4, noted for the participants as "neither relevant nor irrelevant"), and set to "0" otherwise. It is therefore important to note that, for the models computed, an estimated value of 0.5 correspond to the chance level. Therefore, significant effects deviated from this value either positively or negatively.

Online Supplementary Material E

Subscale	Statistical test	
	Median	Z test
Arousal	Mdn (musician) = 4, Mdn (control) = 4	Z = 1.14, p = 0.253
Valence	Mdn (musician) = 1, Mdn (control) = 1	Z = 0.5, p = 0.62
JoyfulActivation	Mdn (musician) = 2, Mdn (control) = 2	Z = 0.74, p = 0.462
Nostalgia	Mdn (musician) = 3, Mdn (control) = 2.5	Z = 1.33, p = 0.183
Peacefulness	Mdn (musician) = 2, Mdn (control) = 2	Z = 1.48, p = 0.14
Power	Mdn (musician) = 2, Mdn (control) = 3	Z = -0.77, p = 0.444
Sadness	Mdn (musician) = 1, Mdn (control) = 2	Z = -1.01, p = 0.315
Tenderness	Mdn (musician) = 2, Mdn (control) = 2	Z = -0.81, p = 0.416
Tension	Mdn (musician) = 1, Mdn (control) = 1	Z = -0.73, p = 0.465
Transcendence	Mdn (musician) = 2, Mdn (control) = 2	Z = 1.52, p = 0.129
Wonder	Mdn (musician) = 3, Mdn (control) = 3	Z = 0.25, p = 0.799
Flow	Mdn (musician) = 3, Mdn (control) = 3	Z = 0.32, p = 0.745
Force	Mdn (musician) = 3, Mdn (control) = 3	Z = -1.2, p = 0.231
Interior	Mdn (musician) = 3, Mdn (control) = 3	Z = -0.8, p = 0.426
Movement	Mdn (musician) = 3, Mdn (control) = 3	Z = -1.07, p = 0.284
Wandering	Mdn (musician) = 3, Mdn (control) = 3	Z = 0.6, p = 0.551
Flow	Mdn (high VVIQ) = 3, Mdn (low VVIQ) = 3	Z = -2.21, p = 0.044
Force	Mdn (high VVIQ) = 3, Mdn (low VVIQ) = 3	Z = -0.41, p = 0.679
Interior	Mdn (high VVIQ) = 3, Mdn (low VVIQ) = 2	Z = -3.25, p = 0.005
Movement	Mdn (high VVIQ) = 3, Mdn (low VVIQ) = 3	Z = -1.63, p = 0.128
Wandering	Mdn (high VVIQ) = 3, Mdn (low VVIQ) = 2	Z = -2.32, p = 0.044

Z test for permutations between two groups within our participants pool.

Online Supplementary Material F

Scale	Subscales	Cronbach Alpha (standardized)
Entrainment	Agitated	0.865
	Animated	0.91
	Beat	0.881
	Dance	0.876
	Entrained	0.865
	Move	0.885
	Physio	0.934
	Resonate	0.904
	Rhythm	0.918
GEMMES	Flow	0.81
	Force	0.849
	Interior	0.791
	Movement	0.875
	Wandering	0.856
GEMS	JoyfulActivation	0.847
	Nostalgia	0.793
	Peacefulness	0.842
	Power	0.895
	Sadness	0.842
	Tenderness	0.838
	Tension	0.827
	Transcendence	0.9
	Wonder	0.858
VA	Arousal	0.904
	Valence	0.703

Reliability of each subscale over the excerpts presented to each participant (Cronbach's alpha)

Online Supplementary Material G

To evaluate if the participants accurately labelled the emotionally connoted excerpts we presented, we performed a GLMM using the binary values. A model encompassing the interaction between the labels participants gave and the labels pre-selected by the music expert, and the main effects associated, statistically outperformed the model with only the main effect (χ^2 (83, N = 81) = 3069.5, p < 0.001, R^2_m = 0.31, R^2_c = 0.44, $AIC_{GEMS*ExcerptGEMS}$ = 13019, $AIC_{GEMS+ExcerptGEMS}$ = 15960; $BIC_{GEMS*ExcerptGEMS}$ = 13640, $BIC_{GEMS+ExcerptGEMS}$ = 16102). Participants gave significantly higher ratings for the specific emotion associated with the excerpt presented (Figure A, Tables A, B, C) (Supplementary Material F, G, H).

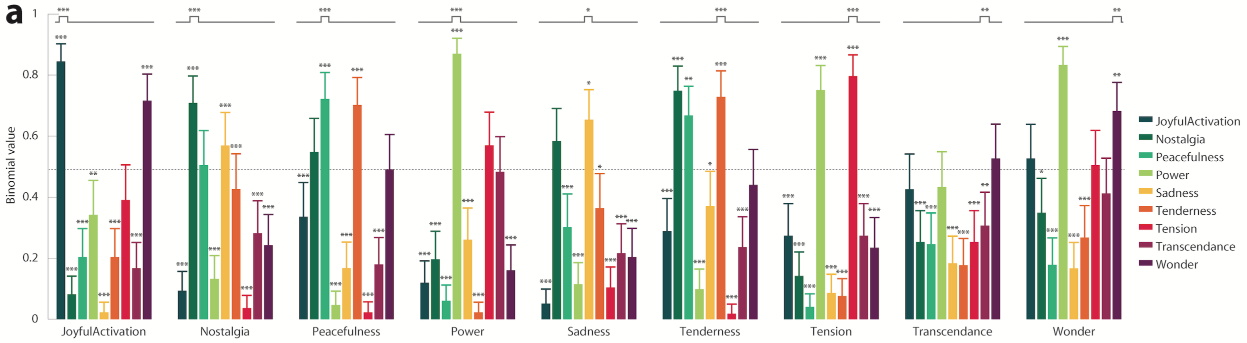

Figure A. **Estimated binary ratings for GEMS based on the attributed affective content of the musical excerpts.** The dotted horizontal line at 0.5 symbolized the chance level of drawing from a binary set. Values are tested to be significantly different from this value. All contrasts are FDR-corrected [*: p < 0.05, **: p < 0.01, ***: p < 0.001]

Excerpts	Joyful Activation	Nostalgia	Peacefulness	Power	Sadness	Tenderness	Tension	Transcendance	Wonder
Joyful Activation	χ^2 = 39.9, p < 0.001	χ^2 = 60.1, p < 0.001	χ^2 = 28.3, p < 0.001	χ^2 = 7.4, p = 0.009	χ^2 = 60.7, p < 0.001	χ^2 = 28.4, p < 0.001	χ^2 = 3.5, p = 0.077	χ^2 = 36.8, p < 0.001	χ^2 = 14.2, p < 0.001
Nostalgia	χ^2 = 56.9, p < 0.001	χ^2 = 13.2, p < 0.001	*ns*	χ^2 = 45.8, p < 0.001	*ns*	*ns*	χ^2 = 65.2, p < 0.001	χ^2 = 14.6, p < 0.001	χ^2 = 20.8, p < 0.001
Peacefulness	χ^2 = 8, p = 0.006	χ^2 = 0.7, p = 0.451	χ^2 = 15, p < 0.001	χ^2 = 65.8, p < 0.001	χ^2 = 36.5, p < 0.001	χ^2 = 12.3, p < 0.001	χ^2 = 59.5, p < 0.001	χ^2 = 33.6, p < 0.001	*ns*
Power	χ^2 = 49.5, p < 0.001	χ^2 = 29.8, p < 0.001	χ^2 = 64.8, p < 0.001	χ^2 = 45.5, p < 0.001	χ^2 = 17.7, p < 0.001	χ^2 = 60.1, p < 0.001	*ns*	*ns*	χ^2 = 38.4, p < 0.001
Sadness	χ^2 = 66.1, p < 0.001	*ns*	χ^2 = 11.9, p < 0.001	χ^2 = 50.7, p < 0.001	χ^2 = 7, p = 0.011	χ^2 = 5.5, p = 0.024	χ^2 = 53.8, p < 0.001	χ^2 = 25.6, p < 0.001	χ^2 = 28.2, p < 0.001
Tenderness	χ^2 = 13.6, p < 0.001	χ^2 = 19.1, p < 0.001	χ^2 = 8.3, p = 0.005	χ^2 = 55.1, p < 0.001	χ^2 = 4.9, p = 0.033	χ^2 = 16, p < 0.001	χ^2 = 55.6, p < 0.001	χ^2 = 21.9, p < 0.001	*ns*
Tension	χ^2 = 15.8, p < 0.001	χ^2 = 43.1, p < 0.001	χ^2 = 66.5, p < 0.001	χ^2 = 19.4, p < 0.001	χ^2 = 58.8, p < 0.001	χ^2 = 61.6, p < 0.001	χ^2 = 27.9, p < 0.001	χ^2 = 15.7, p < 0.001	χ^2 = 22.2, p < 0.001
Transcendance	*ns*	χ^2 = 18.9, p < 0.001	χ^2 = 20, p < 0.001	*ns*	χ^2 = 32.8, p < 0.001	χ^2 = 34.2, p < 0.001	χ^2 = 18.9, p < 0.001	χ^2 = 11.2, p = 0.001	*ns*
Wonder	*ns*	χ^2 = 6.7, p = 0.012	χ^2 = 33.8, p < 0.001	χ^2 = 36.2, p < 0.001	χ^2 = 36.8, p < 0.001	χ^2 = 16.6, p < 0.001	*ns*	*ns*	χ^2 = 9.9, p = 0.002

Table A. **Contrast between each emotional sub-scale and the chance level (0.5) on each emotion represented by the selected excerpt**. A significant difference represents a value above or below 0.5, the chance level in a binomial distribution. All contrasts are FDR-corrected [ns: non-significant]

		Against all
Excerpts	Joyful Activation	$\chi^2 = 39.9$, $p < 0.001$
	Nostalgia	$\chi^2 = 13.2$, $p < 0.001$
	Peacefulness	$\chi^2 = 15$, $p < 0.001$
	Power	$\chi^2 = 45.5$, $p < 0.001$
	Sadness	$\chi^2 = 7$, $p = 0.011$
	Tenderness	$\chi^2 = 16$, $p < 0.001$
	Tension	$\chi^2 = 27.9$, $p < 0.001$
	Transcendance	$\chi^2 = 11.2$, $p = 0.001$
	Wonder	$\chi^2 = 9.9$, $p = 0.002$

Table B. **Contrasts between the emotional sub-scale corresponding to the emotion portrayed in the excerpts and all the other subscales for the same excerpts taken together.** All contrasts are FDR-corrected.

		Joyful Activation	Nostalgia	Peacefulness	Power	Sadness	Tenderness	Tension	Transcendance	Wonder
Principal emotion	Joyful Activation		$\chi^2 = 147.4$, $p < 0.001$	$\chi^2 = 112$, $p < 0.001$	$\chi^2 = 73.2$, $p < 0.001$	$\chi^2 = 119.4$, $p < 0.001$	$\chi^2 = 111.9$, $p < 0.001$	$\chi^2 = 61.4$, $p < 0.001$	$\chi^2 = 123.7$, $p < 0.001$	$\chi^2 = 7.6$, $p = 0.008$
	Nostalgia	$\chi^2 = 103.5$, $p < 0.001$		$\chi^2 = 12.3$, $p < 0.001$	$\chi^2 = 92.3$, $p < 0.001$	$\chi^2 = 6$, $p = 0.018$	$\chi^2 = 22.7$, $p < 0.001$	$\chi^2 = 101.9$, $p < 0.001$	$\chi^2 = 50.6$, $p < 0.001$	$\chi^2 = 60.3$, $p < 0.001$
	Peacefulness	$\chi^2 = 41.7$, $p < 0.001$	$\chi^2 = 9.4$, $p = 0.003$		$\chi^2 = 108.9$, $p < 0.001$	$\chi^2 = 85.2$, $p < 0.001$	*ns*	$\chi^2 = 91$, $p < 0.001$	$\chi^2 = 81.5$, $p < 0.001$	$\chi^2 = 15.9$, $p < 0.001$
	Power	$\chi^2 = 143.7$, $p < 0.001$	$\chi^2 = 119.3$, $p < 0.001$	$\chi^2 = 152.5$, $p < 0.001$		$\chi^2 = 100.2$, $p < 0.001$	$\chi^2 = 122.5$, $p < 0.001$	$\chi^2 = 32.2$, $p < 0.001$	$\chi^2 = 47.8$, $p < 0.001$	$\chi^2 = 130.9$, $p < 0.001$
	Sadness	$\chi^2 = 95.4$, $p < 0.001$	*ns*	$\chi^2 = 34.7$, $p < 0.001$	$\chi^2 = 83.4$, $p < 0.001$		$\chi^2 = 23.6$, $p < 0.001$	$\chi^2 = 86.6$, $p < 0.001$	$\chi^2 = 54.2$, $p < 0.001$	$\chi^2 = 57.6$, $p < 0.001$
	Tenderness	$\chi^2 = 53.9$, $p < 0.001$	*ns*	*ns*	$\chi^2 = 107.1$, $p < 0.001$	$\chi^2 = 36.2$, $p < 0.001$		$\chi^2 = 84.4$, $p < 0.001$	$\chi^2 = 67.1$, $p < 0.001$	$\chi^2 = 24$, $p < 0.001$
	Tension	$\chi^2 = 74.9$, $p < 0.001$	$\chi^2 = 114.1$, $p < 0.001$	$\chi^2 = 125.7$, $p < 0.001$	*ns*	$\chi^2 = 129.2$, $p < 0.001$	$\chi^2 = 130.7$, $p < 0.001$		$\chi^2 = 74.9$, $p < 0.001$	$\chi^2 = 85.8$, $p < 0.001$
	Transcendance	$\chi^2 = 4.4$, $p = 0.046$	*ns*	*ns*	$\chi^2 = 4.9$, $p = 0.034$	$\chi^2 = 6.3$, $p = 0.015$	$\chi^2 = 7$, $p = 0.011$	*ns*		$\chi^2 = 14$, $p < 0.001$
	Wonder	$\chi^2 = 7.2$, $p = 0.01$	$\chi^2 = 30.9$, $p < 0.001$	$\chi^2 = 71.3$, $p < 0.001$	$\chi^2 = 9.4$, $p = 0.003$	$\chi^2 = 74.9$, $p < 0.001$	$\chi^2 = 47.8$, $p < 0.001$	$\chi^2 = 9.2$, $p = 0.004$	$\chi^2 = 20.5$, $p < 0.001$	

Table C. **Contrast between the emotional sub-scale corresponding to the emotion portrayed in the excerpts and each of the other subscales for the same excerpts**. All contrasts are FDR-corrected [ns: non-significant]

Online Supplementary Material H

	Flow	Force	Interior	Movement	Wandering
JoyfulActivation	$\chi^2 = 7.3$, $p = 0.012$	ns	$\chi^2 = 23$, $p < 0.001$	$\chi^2 = 15.4$, $p < 0.001$	*ns*
Nostalgia	*ns*	$\chi^2 = 20.1$, $p < 0.001$	*ns*	$\chi^2 = 12$, $p = 0.001$	$\chi^2 = 14.9$, $p < 0.001$
Peacefulness	$\chi^2 = 15.4$, $p < 0.001$	$\chi^2 = 36.1$, $p < 0.001$	*ns*	$\chi^2 = 14.9$, $p < 0.001$	*ns*
Power	$\chi^2 = 30.1$, $p < 0.001$	$\chi^2 = 40.9$, $p < 0.001$	*ns*	*ns*	$\chi^2 = 6.7$, $p = 0.016$
Sadness	*ns*	$\chi^2 = 17.6$, $p < 0.001$	$\chi^2 = 7.1$, $p = 0.013$	$\chi^2 = 25.5$, $p < 0.001$	$\chi^2 = 12.9$, $p < 0.001$
Tenderness	$\chi^2 = 15.4$, $p < 0.001$	$\chi^2 = 16.5$, $p < 0.001$	*ns*	$\chi^2 = 7.3$, $p = 0.012$	$\chi^2 = 3.9$, $p = 0.071$
Tension	$\chi^2 = 28.6$, $p < 0.001$	$\chi^2 = 22.9$, $p < 0.001$	$\chi^2 = 10.1$, $p = 0.003$	$\chi^2 = 20.8$, $p < 0.001$	*ns*
Transcendance	$\chi^2 = 10.5$, $p = 0.002$	*ns*	*ns*	*ns*	*ns*
Wonder	$\chi^2 = 4.6$, $p = 0.05$	$\chi^2 = 22.7$, $p < 0.001$	$\chi^2 = 23.9$, $p < 0.001$	$\chi^2 = 33.5$, $p < 0.001$	$\chi^2 = 6$, $p = 0.023$

Contrast between each metaphor sub-scale and the chance level (0.5) on each emotion represented by the selected excerpt. A significant difference represents a value above or below 0.5, the chance level in a binomial distribution. All contrasts are FDR-corrected [ns: non-significant];

Online Supplementary Material I

	ChiSq [$\chi^2(12, N = 81)$]	AIC (Interaction)	AIC (Main)	BIC (Interaction)	BIC (Main)
Acous_PC1	$\chi^2 = 19.3$, $p < 0.001$	9189.5	9200.8	9272.1	9255.9
Acous_PC2	$\chi^2 = 185.9$, $p < 0.001$	9015.1	9192.9	9097.7	9248
Entrain_PC1	$\chi^2 = 1353$, $p < 0.001$	7844.5	9189.5	7927	9244.6
Midlev_PC1	$\chi^2 = 124.3$, $p < 0.001$	9084.3	9200.6	9166.8	9255.6
Midlev_PC2	$\chi^2 = 588.8$, $p < 0.001$	8620.4	9201.2	8703	9256.3
JoyfulActivation	$\chi^2 = 389.7$, $p < 0.001$	8814	9195.7	8896.6	9250.7
Nostalgia	$\chi^2 = 725.9$, $p < 0.001$	8481.4	9199.3	8564	9254.4
Peacefulness	$\chi^2 = 848.2$, $p < 0.001$	8358.1	9198.3	8440.7	9253.3
Power	$\chi^2 = 1028.9$, $p < 0.001$	8171.7	9192.5	8254.2	9247.6
Sadness	$\chi^2 = 396$, $p < 0.001$	8810.3	9198.3	8892.9	9253.4
Tenderness	$\chi^2 = 492.7$, $p < 0.001$	8716.2	9201	8798.8	9256
Tension	$\chi^2 = 921.1$, $p < 0.001$	8286.6	9199.6	8369.1	9254.7
Transcendence	$\chi^2 = 282.2$, $p < 0.001$	8927.4	9201.6	9010	9256.6
Wonder	$\chi^2 = 204.2$, $p < 0.001$	9001.4	9197.6	9084	9252.6
Arousal	$\chi^2 = 1010.1$, $p < 0.001$	8192.9	9195	8275.5	9250
Valence	$\chi^2 = 257.3$, $p < 0.001$	8946.8	9196.1	9029.4	9251.1

Chi-squared test comparing each model estimating the binomial value of the GEMMES scale when looking at each item of the other scales. The first model comprises the interaction between a subscale and the metaphor scale, in order to predict the metaphors' values. The second model only contains the main effect and no interaction. All contrasts are FDR-corrected.

Online Supplementary Material J

	Flow	Force	Interior	Movement	Wandering
Acous_PC1	ns	$\chi^2 = 6.5$, $p = 0.016$	ns	ns	ns
Acous_PC2	ns	$\chi^2 = 61.4$, $p < 0.001$	ns	$\chi^2 = 23.4$, $p < 0.001$	$\chi^2 = 10.4$, $p = 0.002$
Entrain_PC1	$\chi^2 = 48.7$, $p < 0.001$	$\chi^2 = 131.5$, $p < 0.001$	$\chi^2 = 57.5$, $p < 0.001$	$\chi^2 = 180$, $p < 0.001$	$\chi^2 = 36.7$, $p < 0.001$
Midlev_PC1	$\chi^2 = 14.6$, $p < 0.001$	$\chi^2 = 9.2$, $p = 0.004$	ns	$\chi^2 = 5.4$, $p = 0.029$	$\chi^2 = 6.1$, $p = 0.02$
Midlev_PC2	$\chi^2 = 15.5$, $p < 0.001$	$\chi^2 = 13$, $p < 0.001$	$\chi^2 = 42$, $p < 0.001$	$\chi^2 = 45.8$, $p < 0.001$	$\chi^2 = 12.1$, $p < 0.001$
JoyfulActivation	ns	$\chi^2 = 23.5$, $p < 0.001$	$\chi^2 = 18.4$, $p < 0.001$	$\chi^2 = 65.3$, $p < 0.001$	$\chi^2 = 22.2$, $p < 0.001$
Nostalgia	$\chi^2 = 34.7$, $p < 0.001$	$\chi^2 = 72.4$, $p < 0.001$	$\chi^2 = 18.5$, $p < 0.001$	$\chi^2 = 41.7$, $p < 0.001$	$\chi^2 = 6$, $p = 0.021$
Peacefulness	$\chi^2 = 44.6$, $p < 0.001$	$\chi^2 = 97.3$, $p < 0.001$	$\chi^2 = 17.1$, $p < 0.001$	$\chi^2 = 54.9$, $p < 0.001$	$\chi^2 = 6.6$, $p = 0.015$
Power	$\chi^2 = 43.1$, $p < 0.001$	$\chi^2 = 180.6$, $p < 0.001$	$\chi^2 = 17.8$, $p < 0.001$	$\chi^2 = 64.9$, $p < 0.001$	$\chi^2 = 11.7$, $p = 0.001$
Sadness	ns	$\chi^2 = 13.8$, $p < 0.001$	$\chi^2 = 25.9$, $p < 0.001$	$\chi^2 = 51.6$, $p < 0.001$	$\chi^2 = 19.8$, $p < 0.001$
Tenderness	$\chi^2 = 31.9$, $p < 0.001$	$\chi^2 = 49.4$, $p < 0.001$	$\chi^2 = 8.4$, $p = 0.006$	$\chi^2 = 19.9$, $p < 0.001$	ns
Tension	$\chi^2 = 44.3$, $p < 0.001$	$\chi^2 = 62.1$, $p < 0.001$	$\chi^2 = 34.2$, $p < 0.001$	$\chi^2 = 81.4$, $p < 0.001$	$\chi^2 = 6.8$, $p = 0.014$
Transcendence	$\chi^2 = 16$, $p < 0.001$	$\chi^2 = 35.6$, $p < 0.001$	$\chi^2 = 6.6$, $p = 0.015$	$\chi^2 = 4.8$, $p = 0.04$	ns
Wonder	ns	ns	$\chi^2 = 13.9$, $p < 0.001$	$\chi^2 = 30.3$, $p < 0.001$	$\chi^2 = 23.2$, $p < 0.001$
Arousal	$\chi^2 = 37.8$, $p < 0.001$	$\chi^2 = 147.8$, $p < 0.001$	$\chi^2 = 23.8$, $p < 0.001$	$\chi^2 = 59.6$, $p < 0.001$	$\chi^2 = 9.6$, $p = 0.003$
Valence	ns	ns	$\chi^2 = 15.3$, $p < 0.001$	$\chi^2 = 50.7$, $p < 0.001$	$\chi^2 = 28.9$, $p < 0.001$

Contrast between the high and low values excerpts on a specific subscale to predict each metaphor subscales. A significant difference represents a different in the metaphor values between excerpts that are labelled as high in one condition and low in the same condition. All contrasts are FDR-corrected [ns: non-significant, *: $p < 0.05$, **: $p < 0.01$, ***: $p < 0.001$]

Online Supplementary Material K

Because every musical metaphor could be characterized by a multitude of different descriptors, ranging from emotions to acoustic and perceptual features, we decided to perform a multi regression with best subset selection to highlight a reduced number of suitable descriptors. A regression with this subset of descriptors should reasonably fit the binomial value for each metaphor. More importantly, the addition of any other descriptors should not improve the fit by a large margin. While running the multi regression analysis, we extracted the Akaike Information Criterion (AIC) for every set of descriptors, starting with one only regressor to finishing with all the regressors in the model. Across all metaphor regressions, adding more than five regressors did not seem to improve the model by a large margin (Figure A). In addition, we observed that the metaphors "Force" and "Movement" were associated with a better fit with the descriptors present in this study than "Flow," "Interior," and "Wandering". Consequently, we looked for the best subset of five regressors to fit each metaphor. While most of the five regressors for each metaphor had a statistically significant impact, some regressors showed a greater weight, meaning that each variation in this parameter resulted in a greater variation in the metaphor itself. To elaborate, we mainly report positive association between "Force" and "Power," "Arousal," and entrainment (Table A). "Movement" was positively with entrainment. "Flow" was positively associated with "Joyful activation", and negatively associated with entrainment. "Interior" was negatively associated with entrainment. Finally, the weights associated with "Wonder" were negative for "Tension" and positive for entrainment.

A potential issue with using multiple regressors to describe metaphors lies in multicollinearities. Some of the descriptors might be strongly correlated and therefore might explain the same part of the variance in our models. To explore that, we decided to compute the individual variance (R^2), the variance inflation factors (VIF), and the correlation matrix. First, not all regressors explains the same amount of variance. Some might explain more of the variance of a certain model when taken individually. For example, the variance associated with the metaphor "Force" is better explained by "Power" ($R^2 = 0.251$), arousal ($R^2 = 0.282$), and entrainment PC1 ($R^2 = 0.191$) than "Wonder" ($R^2 = 0.005$) despite all of these regressors being in the best subset (Table B). "Movement" is best explained by entrainment PC1 ($R^2 = 0.259$). Second, not all regressors share the variance explained. The variance inflation factor shows which regressor is linked with others, with values approaching 1 when the regressor is independent of other regressors. In our data, some were characterizing the same variance. It is the case, for example, for "Tenderness" and "Nostalgia" in the "Movement" model, with very high VIF (respectively 22.08 and 22.19). "Joyful activation" and entrainment PC1 seemed to be sharing a part of the variance, with VIF = 3.48 and 4.45 respectively.

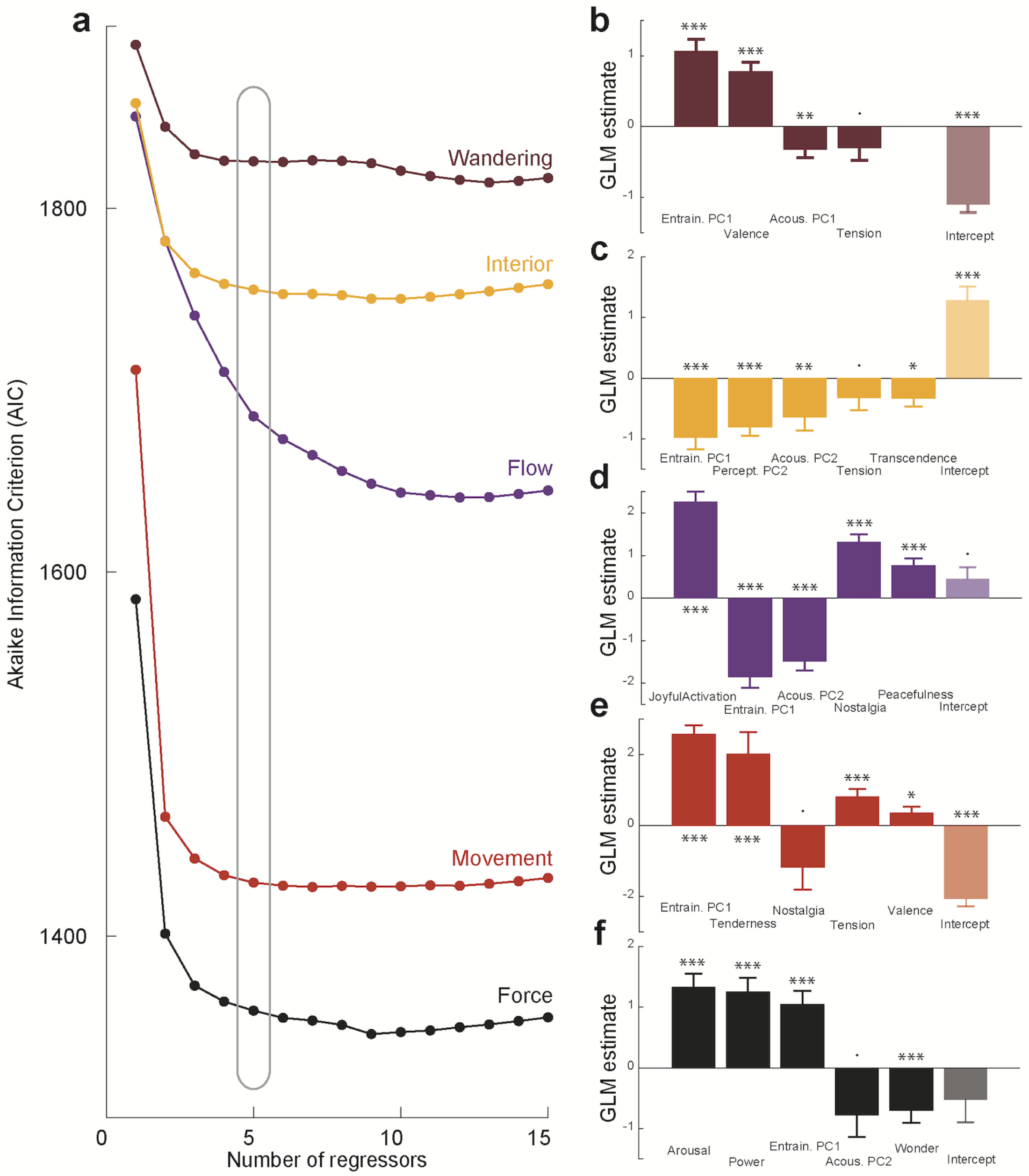

Figures A, B, C, D, E, F. **Multi regression using best subset selection. A.** Evolution of Akaike Information Criterion associated with models with increasing subset sizes. **B.** Weights associated with the best subset of regressors to estimate the binomial value of the metaphor "Wandering". **C.** Weights associated with the best subset of regressors to estimate the binomial value of the metaphor "Interior". **D.** Weights associated with the best subset of regressors to estimate the binomial value of the metaphor "Flow". **E.** Weights associated with the best subset of regressors to estimate the binomial value of the metaphor "Movement". **F.** Weights associated with the best subset of regressors to estimate the binomial value of the metaphor "Force". The faded bar represents the intercept present in every regression. [ns: non-significant, *: $p < 0.05$, **: $p < 0.01$, ***: $p < 0.001$]

Metaphor	Regressor	Estimate	Statistique
Flow	(Intercept)	0.454	z = 1.674, p = 0.094
	JoyfulActivation	2.26	z = 9.482, p < 0.001
	Nostalgia	1.326	z = 7.598, p < 0.001
	Peacefulness	0.768	z = 4.615, p < 0.001
	Acous. PC2	-1.48	z = -6.586, p < 0.001
	Entrain. PC1	-1.855	z = -7.293, p < 0.001
Force	(Intercept)	-0.519	z = -1.38, p = 0.168
	Power	1.252	z = 5.498, p < 0.001
	Wonder	-0.705	z = -3.462, p < 0.001
	Arousal	1.335	z = 6.16, p < 0.001
	Acous. PC2	-0.778	z = -2.175, p = 0.03
	Entrain. PC1	1.046	z = 4.834, p < 0.001
Interior	(Intercept)	1.275	z = 5.552, p < 0.001
	Tension	-0.328	z = -1.661, p = 0.097
	Transcendence	-0.33	z = -2.428, p = 0.015
	Acous. PC2	-0.646	z = -2.978, p = 0.003
	Entrain. PC1	-0.975	z = -4.893, p < 0.001
	Percept. PC2	-0.806	z = -5.856, p < 0.001
Movement	(Intercept)	-2.068	z = -9.787, p < 0.001
	Nostalgia	-1.178	z = -1.884, p = 0.06
	Tenderness	2.031	z = 3.34, p < 0.001
	Tension	0.822	z = 3.908, p < 0.001
	Valence	0.36	z = 2.097, p = 0.036
	Entrain. PC1	2.586	z = 10.369, p < 0.001
Wandering	(Intercept)	-1.093	z = -9.577, p < 0.001
	Tension	-0.302	z = -1.754, p = 0.079
	Valence	0.776	z = 5.748, p < 0.001
	Acous. PC1	-0.321	z = -2.744, p = 0.006
	Entrain. PC1	1.065	z = 6.372, p < 0.001

Table A. **Coefficient estimates and significant level when compared to zero for each regression with best subset**. [ns: non-significant, *: p < 0.05, **: p < 0.01, ***: p < 0.001]

		Statistics	
Model	Condition	R^2	Variance inflation factors
Flow	Joyful activation	0.006	3.48
	Entrainment PC1	0.072	4.45
	Acoustic PC2	0.001	1.33
	Nostalgia	0.068	2.16
	Peacefulness	0.082	1.87
Force	Arousal	0.251	2.22
	Power	0.282	2.38
	Entrainment PC1	0.191	2.43
	Acoustic PC2	0.089	1.14
	Wonder	0.005	2.07
Interior	Entrainment PC1	0.085	2.79
	Perceptual PC2	0.085	1.34
	Acoustic PC2	0.001	1.42
	Tension	0.063	2.02
	Transcendence	0.013	1.04
Movement	Entrainment PC1	0.259	3.49
	Tenderness	0.04	21.08
	Nostalgia	0.081	22.19
	Tension	0.149	1.85
	Valence	0.078	1.31
Wandering	Entrainment PC1	0.055	2.2
	Valence	0.045	1.17
	Acoustic PC1	0.003	1.02
	Tension	0.013	2

Table B. **Multicollinearities in form of variance inflation factors and variances explained in term of R^2 for each regressor in each regression with best subset.**

Online Supplementary Material L

Cluster	Distance	Silhouette
2	14.02	0.55
3	9.8	0.51
4	7.8	0.47
5	6.23	0.53
**6**	**4.94**	**0.52**

Computed mean distance between the barycenter of each cluster and the constituent items as well as mean silhouette value for each cluster. The cluster analysis conducted was K-mean with city block.

Online Supplementary Material M

The prevalence of zero ratings in our dataset can be explained by multiple reasons. The first one is associated with the way the scales are designed. While being continuous scales, both GEMS and GEMMES feel like categorical scales. Even if music is capable of eliciting mixed emotions (Juslin et al., 2011), participants, in our study, tend to choose very few or even sometimes only one category to represent one musical piece. This kind of behavior was already observed in the study leading to the creation of the GEMMES (Schaerlaeken et al., 2019). The way both GEMMES and GEMS were created, by allowing rotation in the confirmatory factorial analysis, resulted in maximizing the discrimination between the different factors (Schaerlaeken et al., 2019; Zentner et al., 2008). The more discriminable the items in a scale, the more zeros we found in the participants responses on the not relevant subscales. It reflects that participants favour some descriptive patterns over others. In contrary, valence and arousal are seen as dimensions and not categories (Russell, 1980), consequently, they invite the participants to rate both independently. Furthermore, the presence of zeros in our dataset might reflect the ineffability of music in general. It has been argued that participants sometimes "lack the necessary vocabulary to provide accurate verbalizations of their emotional experience" (Zentner and Eerola (2010), p. 193)). People also tend to categorize their subjective experience rather than representing them in a more complex mixed feeling. Evaluating the emotional content of one entire excerpt at once rather than dynamically over the course of the piece may also bias the listener's judgment. Actually, it has been shown that this integration process changes a lot the reported emotions compared to dynamic judgments for the same excerpts (Eliard, 2017). Furthermore, some subscales are used less frequently than others (e.g. "Transcendence" and "Wonder"), as they lead to more zeros in the data collected (Aljanaki, Wiering, & Veltkamp, 2016). This might be related to the fact that people are less used to represent these categories, or that these categories are ill-defined, or that these categories are more rarely induced than others.

References Online Supplementary Material L

Juslin, P. N., Liljestrom, S., Laukka, P., Vastfjall, D., & Lundqvist, L.-O. (2011). Emotional reactions to music in a nationally representative sample of Swedish adults: Prevalence and causal influences. *Musicae Scientiae*, *15*(2), 174–207

Schaerlaeken, S., Glowinski, D., Rappaz, M.-A., & Grandjean, D. (2019). “hearing music as...”: Metaphors evoked by the sound of classical music. *Psychomusicology: Music, Mind, and Brain*, *29*(2-3), 100.

Zentner, M., Grandjean, D., & Scherer, K. R. (2008, aug). Emotions evoked by the sound of music: characterization, classification, and measurement. *Emotion (Washington, D.C.)*, *8*(4), 494–521.

Russell, J. A. (1980). A circumplex model of aect. *Journal of personality and social psychology*, *39*(6), 1161

Zentner, M., & Eerola, T. (2010). Self-report measures and models. *Handbook of music and emotion*, 187–221.

Eliard, K. (2017). *Dynamiques temporelles des emotions exprimees par la musique* (Unpublished doctoral dissertation). University of Geneva.

Aljanaki, A., Wiering, F., & Veltkamp, R. C. (2016). Studying emotion induced by music through a crowdsourcing game. *Information Processing & Management*, *52*(1), 115–128.
